# Media’s role in agenda-setting: Exploring media coverage of the German Baltic fishery discourse

**DOI:** 10.1007/s13280-026-02371-5

**Published:** 2026-03-25

**Authors:** Jasper Bär, Annegret Kuhn, Kai Carstensen, Heike Schwermer

**Affiliations:** 1 Institute for Statistics and Econometrics, Christian-Albrechts-Universität zu Kiel, Wilhelm-Seelig-Platz 7, 24118 Kiel, Germany; 2 Center for Ocean and Society, Christian-Albrechts-Universität zu Kiel, Fraunhoferstraße 16, 24118 Kiel, Germany

**Keywords:** Agenda-setting, Baltic Sea, Fisheries management, Media, Sentiment analysis, Stakeholder analysis

## Abstract

In this paper, we explore how media covers and frames fishery issues. Focusing on the German Baltic fishery, we use methods from computational linguistics, political science, and fisheries science to analyze almost 1800 newspaper articles from 2009 to 2021. We document four key results. First, political events—rather than scientific policy advice—drive media reporting. Second, fishery representatives are mentioned more often than environmental non-governmental organizations (eNGOs) and with a much higher level of personalization and emotionalization. Third, reductions in European Union (EU) fishing quotas are followed by negative media sentiment, even though the status of the Baltic ecosystem would improve. Fourth, media portrayal of relevant stakeholder groups is consistent with the existence of two opposing coalitions: one emphasizing the socio-economic viability of the fishery and one advocating for the ecosystem’s ecological health. Overall, we find evidence that newspapers tend to act as “advocates” of the fishers’ interests and concerns.

**Supplementary Information:**

The online version contains supplementary material available at https://doi.org/10.1007/s13280-026-02371-5.

## Introduction

### The Baltic Sea fisheries crisis

The German Baltic Sea and its fisheries are in a serious crisis. Multifaceted anthropogenic impacts such as climate change, eutrophication, and the overexploitation of marine resources have led to the marine ecosystem of the Baltic Sea being severely threatened and its fisheries sector undergoing sharp structural changes (de Graaf et al. 2023). While fish species diversity in the Baltic Sea has always been low compared to other marine ecosystems, the last decade has witnessed an increasingly strong reduction in the spawning stock biomasses of its most commercially relevant species, cod (*Gadus morhua*) and herring (*Clupea harengus*), see Möllmann et al. (2021), Moll et al. (2024) and ICES (2025a, b). The associated severe limitations on target fish species have intensified conflicts over resources and space between various stakeholder groups (Schwermer et al. 2021).

### The role of media in public discourse

Media, especially newspapers (both print and online), serve as essential channels informing the public about political and societal processes, thus shaping public discourse and influencing policy agendas (Wilson 1995; Vliegenthart et al. 2016; Hansen 2018; James et al. 2019). In fact, recent research underscores that traditional news media remain highly influential, especially in contexts characterized by diverse stakeholder groups as well as complex and multi-layered regulatory frameworks like those found in European Union (EU) fisheries governance and management (EU 2013; Kennedy and Prat 2019; Carvalho et al. 2025). The frequency and content of media coverage substantially determine the information available to the public, thereby affecting public perception and understanding of fisheries management decisions (Condie et al. 2022).

However, research on media coverage of fisheries issues is limited. Most existing studies focus specifically on aquaculture (Olsen and Osmundsen 2017; Duffy et al. 2019; Govaerts 2021), and often emphasize the amount or frequency of media coverage without exploring qualitative dimensions, such as the tone. As a result, detailed insights into patterns and implications of media reporting on fisheries management are scarce. Jönsson’s (2011) foundational analysis of media coverage related to Baltic Sea environmental issues remains influential but predates the EU Common Fisheries Policy reform (EU 2013) and the subsequent escalation of the Baltic's ecological crisis. Recent research by Nilsson and Sandström (2024) examines stakeholder coalitions within the Swedish eel fishery debate, highlighting how advocacy coalitions are represented in selected media reports and public consultations. While their analysis offers valuable insights, it focuses on eel fishery in Sweden and uses a qualitative methodology based on a limited sample of newspaper articles. This highlights the need for a broader, quantitative investigation into media coverage of Baltic fisheries.

This paper contributes to the literature by offering a comprehensive analysis of the media's role in the German Baltic fishery, applying methods from computational linguistics and machine learning. Specifically, we classify the sentiment, identify key topics, and perform a stance classification that distinguishes between socio-economic and ecological arguments to systematically examine a large corpus of nearly 1800 German newspaper articles related to the German Baltic fishery, published between 2009 and 2021.

### Methodology and data

To guide our analysis, we employ agenda-setting theory (McCombs and Shaw 1972). This theory distinguishes between two complementary levels of media influence. According to first-level agenda-setting theory, the amount and frequency of news coverage of a topic influence the extent to which the public considers it to be important; in other words, it focuses on what the media reports on. Second-level agenda-setting theory is centered on the attributes of an object or person and is linked to how the media reports (Kim and McCombs 2007).

Drawing on agenda-setting theory, this paper answers the following overarching research question: How do German newspapers cover and frame the German Baltic fishery and its policy debates? We examine four dimensions of media influence. At the first level of agenda-setting theory, we analyze (i) timing and amount—when and how often do newspapers report on Baltic fishery issues?—and (ii) stakeholder voice—which stakeholders have a voice in the media? At the second level, we explore (iii) sentiment—in which tone is the German Baltic fishery portrayed and does this tone change over time and across stakeholder groups?—and (iv) stakeholder stance and focus—how do the media represent the stakeholders’ positions towards the German Baltic fishery and which topics do they emphasize?

Our analytical approach consists of three sequential steps. First, we measure media attention by counting fishery‐related articles by month. Second, we perform a sentiment analysis utilizing BERT (Bidirectional Encoder Representations from Transformers, Devlin et al. 2019) to label every sentence in our dataset as positive, neutral, or negative, and then aggregate these labels into quarterly sentiment indices. Third, we conduct a stakeholder analysis by extracting all named individuals and organizations using a named entity recognition model by Schweter and Akbik (2020), assigning them to one of four groups (Fishery, environmental non-governmental organizations (eNGOs), Science, and Politics & Public Authorities), and for each group computing its media salience (mentions per article), overall sentiment, fishery‐stance index (share of arguments focused on socio-economic viability versus ecological health), and the distribution of topics they address.

Why do we focus on newspapers? While social media such as X (formerly Twitter) and Instagram have become increasingly popular in recent years (Fletcher and Nielsen 2018; Gilardi et al. 2022; Stoddart et al. 2025), newspapers—both online and in print—remain one of the most important sources of information (Kennedy and Prat 2019) and thereby shape public discourses (James et al. 2019). In addition, the social media landscape is relatively new and evolving swiftly which is why social media data are not well-suited for analyzing long time series, as we do in our study.

### Political and social importance of German Baltic Sea fisheries

Why do we analyze the German Baltic fishery? The German Baltic fishery has recently attracted high attention at both societal and political levels, particularly following the publication of the final report by the governmental Commission for Developing a Guiding Vision for the Future of the Baltic Sea Fisheries (BMEL 2023). The establishment of this commission can be understood as a political reaction to the ongoing structural change of the German Baltic fishery, driven both by ecological factors (e.g., the decline in target fish species) and socio-economic pressures (e.g., the increase in fuel prices). Concretely, the number of fishing businesses and vessels has been declining for many years, the sector struggles to attract young entrants, and the closure of producer organizations and ports has eroded essential infrastructure for fishing. This, in turn, has led to sharp price increases for services such as fish transport.

While the overall situation of fisheries is similar in other Baltic states, the public and political discourses vary due to different national management measures—such as Individual Transferable Quotas (ITQs) —and region-specific ecological impacts like high abundance of grey seals (Hoff et al. 2021; Svels et al 2025). Against this background, we focus on the German Baltic Sea fishery. Nevertheless, we believe that the policy recommendations derived from this case may be at least partly transferable to other Baltic fisheries as all are regulated under the umbrella of the European Common Fisheries Policy (CFP), which is of prime importance for both the fisheries and the marine ecosystem (EU 2013).

### EU fisheries governance and management

The CFP aims to ensure the sustainable management of the European fishing fleets and the conservation of fish stocks. It includes measures regulating *how much* (e.g. Borges 2021), *where* (e.g. Funk et al. 2020; Kriegl et al. 2021) and *when* (e.g. Berkström et al. 2021; Rufener et al. 2023) fish may be caught. A key element of the CFP—central to this paper—is the negotiation and allocation of fishing quotas. These quotas are based on scientific advice provided by the International Council for the Exploration of the Sea (ICES). Based on this advice, the EU Commission drafts a proposal for annual fishing quotas, which is then submitted to the European Council for adoption and publication (EU 2024; ICES 2025a,b). The fishing quotas are allocated to its member states according to the principle of relative stability, a historically contested issue (Baudron et al. 2020; Morin 2000).

In addition to quota allocations, other European policy frameworks—such as the EU Marine Strategy Framework Directive (EU 2008) and the EU Biodiversity Strategy (EU 2022)—also affect fisheries and marine ecosystems. These overlapping frameworks create divergent agendas among stakeholders (Schwermer et al. 2021; Nilsson and Sandström 2024): fishers’ associations advocate for quota stability and socio‐economic support, while eNGOs press for stronger conservation measures. Scientists aim to translate their assessments into policy (ICES 2025a,b), and public authorities must balance these competing demands. Because newspapers remain a primary arena for these debates (Wilson 1995; James et al. 2019), analyzing media coverage is crucial for understanding how policy outcomes are shaped. Accordingly, our findings are highly relevant not only for the future development of the German Baltic Sea fishery, but also for the European fisheries policy more broadly.

## Literature review and theoretical framework

A widely used concept for analyzing the relationship between media, public discourses and public opinion is agenda-setting, a term strongly influenced by the work of McCombs (McCombs and Shaw 1972; McCombs and Reynolds 2002; McCombs 2005; Sheafer 2007). The concept refers to the media’s capacity to influence which topics are on the public agenda. McCombs and Shaw (1972) demonstrated that the amount (how much) and frequency (how often) of news coverage—referred to as first-level agenda-setting—shape public perceptions of issue importance. Building on this, Kim and McCombs (2007) and Sheafer (2007) introduced a second level of agenda-setting which focuses on the attributes of coverage. Kim and McCombs (2007) further distinguish between two groups of attributes for second-level agenda-setting: substantive attributes describe how an object is defined, while affective attributes refer to the tone (positive, neutral, or negative) in which an object is presented in the media (Sheafer 2007). According to second-level agenda-setting theory, both dimensions are of significant importance as media framing influences public perception. For example, in substantive terms, it makes a difference whether the Baltic Sea fishery is mostly portrayed as the cause of an environmental crisis, a victim of overly restrictive quotas, or a guardian of maritime heritage.

A complementary line of research focuses on the effects of personalized and emotional storytelling, indicating that such narratives foster readers’ identification and empathy with the concerns and interests of the principal subjects (Glaser et al. 2009; Fernandez-Quintanilla 2020). The broader effects of such affective identification are diverse and have been analyzed across different disciplines, including electoral research in political science (Mughan 2000), research on knowledge acquisition in science education (Glaser et al. 2009), and communication and media studies (Schramm and Wirth 2006). Despite disciplinary differences, there is a general consensus that affective identification significantly shapes human perception and behavior.

Most existing empirical research in the environmental domain primarily emphasizes first-level agenda-setting, often in combination with substantive framing, while affective attributes tend to be underrepresented (Holt and Barkenmeyer 2012; Mercado 2012). One attribute-oriented study by Hallgren et al. (2020), examining Swedish media coverage, reveals that livestock farmers are depicted with fluctuating roles—as environmental heroes in some narratives and as villains in others—particularly as environmental policy expectations shift.

While agricultural and environmental media representation has received considerable scholarly attention, fisheries-related issues remain comparatively understudied. Most available studies focus primarily on aquaculture (Amberg and Hall 2008; Froehlich et al. 2017; Condie et al. 2022). For example, Froehlich et al. (2017) systematically apply sentiment analysis to aquaculture headlines across multiple countries, observing a generally positive global trend but noting a distinctly negative tone in developed nations, especially regarding marine and offshore aquaculture. Similarly, Condie et al. (2022) utilize an agenda-setting lens explicitly to analyze the long-term media discourse around aquaculture expansion in Tasmania, Australia, demonstrating how media coverage has shaped public awareness and contributed to polarized public debates.

In Germany in particular, marine-related media analyses remain scarce. Feucht and Zander (2017) highlight that German newspaper coverage of aquaculture tends to emphasize economic benefits, often downplaying environmental concerns. Pechmann and Hinkel (2025), analyzing coverage of the proposed Baltic Sea national park, note competing frames where marine ecosystems and local communities are alternately portrayed as vulnerable entities deserving protection.

The studies most closely related to ours are those of Jönsson (2011) and Nilsson and Sandström (2024). Jönsson (2011) examines media representation of environmental risks in the Baltic Sea using Swedish newspapers. Like our study, Jönsson's work includes a systematic analysis of which groups of actors are quoted, thereby determining who is publicly heard—and who is rather not heard. Further, eutrophication is portrayed as the most significant threat in the media, primarily framed by public authorities, but the risk of overfishing also received substantial coverage (Jönsson 2011). Unlike Jönsson's study, our empirical analysis does not focus solely on environmental risk associated with fisheries. Instead, we examine how German newspapers represent fisheries as a whole. Furthermore, our 2009–2021 newspaper corpus allows us to analyze the media discourse during a period of critical transformation, capturing the response to the 2013 Common Fisheries Policy reform and the subsequent, escalating crisis for key fish stocks.

Nilsson and Sandström (2024) focus on the formation of opposing stakeholder coalitions in the Swedish eel fishery debate. Using policy documents and statements from public consultation processes, they find that one coalition champions the interests of the eel fishing industry, emphasizing its economic significance and cultural heritage. An opposing coalition, meanwhile, advocates for stronger environmental protection measures and sustainable fishing practices to address concerns such as overfishing and ecological degradation. They verify the existence of these two coalitions through qualitative newspaper analysis, contributing to our understanding of how such advocacy coalitions are reflected in the Swedish media. We complement this type of evidence with a more quantitative approach, utilizing a larger set of articles and offering a finer-grained analysis of various stakeholder groups.

## Materials and methods

### Dataset

To create a comprehensive database for our analysis of the discourse on the German Baltic fishery system, we collected German national and regional newspaper articles dealing with the following topics: (i) the German Baltic Sea fisheries, (ii) the Baltic Sea ecosystem including its system components, their interactions and dynamics, and (iii) the (administrative as well as political) management of its marine resources including the regulation of harvesting fish and environmental protection. We consider the period from 2009 to 2021 as the digitalization of news media resulted in a significant increase in the availability of online articles after 2009.

To collect articles relevant to our paper, we proceeded in two steps. In the first step, we applied a keyword search using the two keywords ‘Ostsee’ (Baltic Sea) and ‘Fischerei’ (fishery). We drew on the following sources: the news aggregation service *LexisNexis*^1^, which provides access to large datasets from various national newspapers, the online archives of several regional newspapers, and Google News. In the second step, we filtered out all articles that had no direct relation to the German Baltic fishery (e.g., the article's focus was on other marine systems, such as the tropics). This step primarily involved reviewing the main headings and sub-introductions of all articles by the first and last authors of this paper. Furthermore, we only kept articles from newspapers that appeared at least three times in our dataset to ensure they represent well-established sources for the German Baltic fishery. Thereby, we collected a total of 1772 articles from 2009 to 2021 (for a detailed list of newspapers, see Table S5 in the Supporting Information).

In our dataset, 11.5% of all articles are created by the largest German news agency, dpa. These articles can be republished by newspapers that pay for the right to do so. As a result, similar dpa-based articles often appear in multiple newspapers on the same day. As Boydstun et al. (2014) note, when the same story erupts across multiple outlets, it creates a sudden surge in news coverage that underscores the issue's broad salience. Hence, rather than discarding such duplicates, we treat them as positive evidence of an issue’s relevance and leave them in our dataset.

### Empirical analysis

Our analysis is structured into three key parts: quantitative analysis, sentiment analysis, and stakeholder analysis, which we describe in the following.

#### Timing and frequency of media reporting

The quantitative analysis addresses first-level agenda-setting by counting when and how often German newspapers report about the German Baltic fishery. As a preliminary result, we note that the number of articles published has increased over time, with a larger proportion of articles originating from more recent years (see Fig. S4 in the Supporting Information). To properly account for the trend and obtain an unbiased picture of the within-year fluctuations in media attention with respect to the German Baltic fishery, we normalized the yearly results of our stakeholder analysis by the total number of articles published each year. Without this normalization, the most recent years with a large number of published articles would disproportionately affect our results.

#### Sentiment analysis of fisheries quota

We employ a supervised model approach, specifically utilizing BERT (Devlin et al. 2019), a model for text classification tasks including sentiment analysis (for details, see Section Text Classification with BERT in the Supporting Information). This approach allows us to analyze a larger number of newspapers compared to manual coding, ensuring impartiality and practicality, especially given the high number of sentences. With this approach, the sentiment analysis can also be easily extended each year, allowing to closely monitor media attention over time.

Sentiment is computed on the sentence level. To train the model, we created a training dataset by taking a subsample of sentences from our full dataset and annotating them as positive, neutral, or negative. To ensure reliability and minimize bias in our annotations, we engaged four annotators—two authors of this paper and two student assistants^2^—to perform the annotation task. From our total sample of approximately 44 000 sentences, we drew 4000 for building the dataset: 2400 were used for training, 800 for validation, and 800 for testing (for details on the annotation process, see Section Annotation Guidelines in the Supporting Information). The quality of the model is reflected by an 88.6% accuracy on the 800 test sentences (see Supporting Information Section Sentiment Classification for further details).

We then classified all 44 000 sentences in our dataset by the BERT model to create a quarterly sentiment index. To this end, we first computed the share of positive, neutral, and negative sentences in the total number of sentences for a particular quarter. We then defined the sentiment index as the difference between the positive and negative shares. For instance, a value of -0.2 for a given quarter means that 20 percentage points more negative than positive sentences are published in this quarter. This methodology aligns with similar sentiment analysis approaches reported in the literature (e.g., Jha et al. 2021; Costola et al. 2023).

To relate sentiment to (species-specific) fishing quota changes, we additionally calculated the sentiment index for cod and herring, the two most important fish species. To this end, we selected only those articles that include the word ‘cod’ or ‘herring’ at least once. Then we computed the difference between the shares of positive and negative species-specific sentences in the total number of species-specific sentences (i.e., sentences in articles related to cod or herring).

#### Analysis of stakeholder frequency and sentiment

To analyze stakeholder representation in newspaper coverage, we used a model developed by Schweter and Akbik (2020) that extracts named individuals and organizations from text. All individuals and organizations were identified and assigned to one of the following four stakeholder groups (Schwermer et al. 2021): “Fisheries” (commercial fishers, their organizations and representatives), “eNGOs” (organizations involved in the environmental protection and conservation of marine ecosystems), “Politics & Public Authorities” (executive and legislative political bodies on European, national and subnational levels, as well as administrative bodies responsible for the implementation of CFP measures), and “Science” (e.g., universities and academic research institutions). Purely subsistence fisheries were not included as a separate group as they play a negligible role in the German Baltic fishery. The relevance of these four stakeholder groups stems from European fisheries management, which primarily requires their involvement in, for example, regional advisory groups (e.g., Baltic Sea Advisory Board, see EU 2013). If only the individuals were named without mentioning the organization they belong to, we manually assigned them to a group after conducting, for example, an internet search.

We extracted all stakeholder organizations and individual stakeholders from our dataset. To concentrate on the most relevant ones, we only considered organizations and individuals mentioned more than 20 times, but we noted that our results were robust to lowering these thresholds (see Section Stakeholder Selection Threshold in the Supporting Information). We then assigned them to one of the four stakeholder groups. Sentences were ascribed to a stakeholder group based on the presence of a stakeholder within a sentence. If a sentence contained stakeholders from multiple groups, it was assigned to each group simultaneously. We used this stakeholder classification to assess four dimensions of interest: stakeholder media salience, stakeholder sentiment, stance, and stakeholder priorities. We describe the construction details in the following.

Stakeholder media salience is the degree of attention the media devote to each stakeholder group. We measured it by counting the yearly newspaper occurrences of the stakeholder names for each stakeholder group and dividing it by the total number of articles in the corresponding year.

Stakeholder sentiment refers to the tone of sentences that mention a certain stakeholder group. We measured it by a sentiment index for each stakeholder group. To this end, we computed the difference between the shares of positive and negative group-specific sentences (i.e., sentences assigned to a specific stakeholder group) in the total number of group-specific sentences.

Stakeholder fishery stance refers to the position taken by a stakeholder group regarding the fisheries. We constructed a fishery stance index for each group and for relevant subgroups, as follows. Using the BERT model, we classified each sentence in our dataset as (a) focused on socio-economic viability, i.e., emphasizing the needs, challenges, and (cultural) importance of the fishery sector, (b) focused on ecological health, highlighting environmental concerns, sustainability risks, or the need for stricter conservation measures, or (c) neutral (for details, see Section Text Classification with BERT in the Supporting Information). We created a training dataset by drawing a subsample of 2,500 sentences from our full corpus and manually annotating each sentence as either focused on socio-economic viability, focused on ecological health, or neutral. One author of this paper and two student assistants performed the annotation task. We then used 1,500 sentences for training, 500 for validation, and 500 for testing. The model achieved an accuracy of 88.5% on the test sentences (see Supporting Information Section Fishery Stance Classification for further details). We finally applied the trained model to classify all sentences in our dataset and used the classified sentences to create a yearly fishery stance index for each stakeholder group. For each year, we first computed the share of group-specific sentences focused on socio-economic viability and the share focused on ecological health and then defined the fishery stance index as the difference between these two shares.

Stakeholder priorities refer to the fishery-related topics the individual stakeholder groups are most concerned with. Based on expert knowledge, we specified five topics that capture the main dimensions of the media debate: regulatory fishery law, scientific (governance) advice, fisheries subsidies and institutional support, environmental and nature conservation, and all others (Table 1). We employed a BERT model to classify each fishery-related sentence in our dataset as belonging to one of the five topics. To train the model, we created a training dataset by drawing a subsample of 3000 sentences from our full corpus and manually assigned each sentence to one of our five categories by several annotators. We then used 1800 sentences for training, 600 for validation, and 600 for testing our model. Our topic classification achieved an accuracy of 74.5% on our test sentences (see Supporting Information 1.4.3 for further details). This is a robust result for a challenging 5-class classification task, where a random baseline would be 20% accuracy. The specialized topics feature overlapping terminology, making the classification inherently more complex than the general sentiment task (88.6% accuracy). After training our model, we classified each sentence according to its topic and calculated the share of sentences belonging to each topic for each stakeholder group.

**Table 1:** Definition of topics and examples. The table defines the five topic categories used for classifying fishery related sentences in the newspaper corpus (2009–2021). For each topic, it provides a short definition and an illustrative example sentence

Topic name	Description	Example
Scientific (governance) advice	Sentences that reference scientific guidance informing fisheries management, i.e. stock assessments, ICES catch-limit advice, and other research-based advice	The International Council for the Exploration of the Sea (ICES) has advised increasing fishing quotas for the western Baltic Sea by six percent for the year 2011, the Association of German Cutter and Coastal Fishermen announced yesterday in Hamburg
Regulatory fishery law	Sentences that discuss the legal and regulatory frameworks governing fisheries, i.e., quotas, gear or season restrictions, licensing requirements, enforcement measures, etc	The European fisheries ministers agreed on next year’s Baltic Sea fishing quotas yesterday, Tuesday, in Luxembourg
Fisheries subsidies and institutional support	Fisheries subsidies (support through investment measures in fisheries businesses, e.g., scrapping bonus) and institutional support (support from, for example, fisheries associations and clubs supporting the fisheries sector through organized activities, e.g., cutter demonstration, fisheries conference) provided to the fisheries sector	The fishers and the EuroBaltic fish factory in Sassnitz expect the Marine Stewardship Council's label to improve the marketability of herring and lead to higher revenues
Environmental and nature conservation	Aspects of environmental sustainability beyond fisheries management, i.e., environmental concerns including habitat protection, biodiversity conservation and impacts of fishing practices on marine ecosystems and its related policies (e.g., Habitats Directive)	According to environmental NGOs, marine protection in the North Sea and Baltic Sea exists only on paper so far
Others	Sentences that do not clearly fit into any of the above categories, including general news reports, human interest stories, historical accounts, or statements without a strong thematic focus on stocks, aid, or sustainability	Federal Minister of Agriculture Ilse Aigner (CSU) is on a summer tour in the northeast of the country

## Results

### Distribution of media articles on German baltic fisheries

**Fig. 1:**
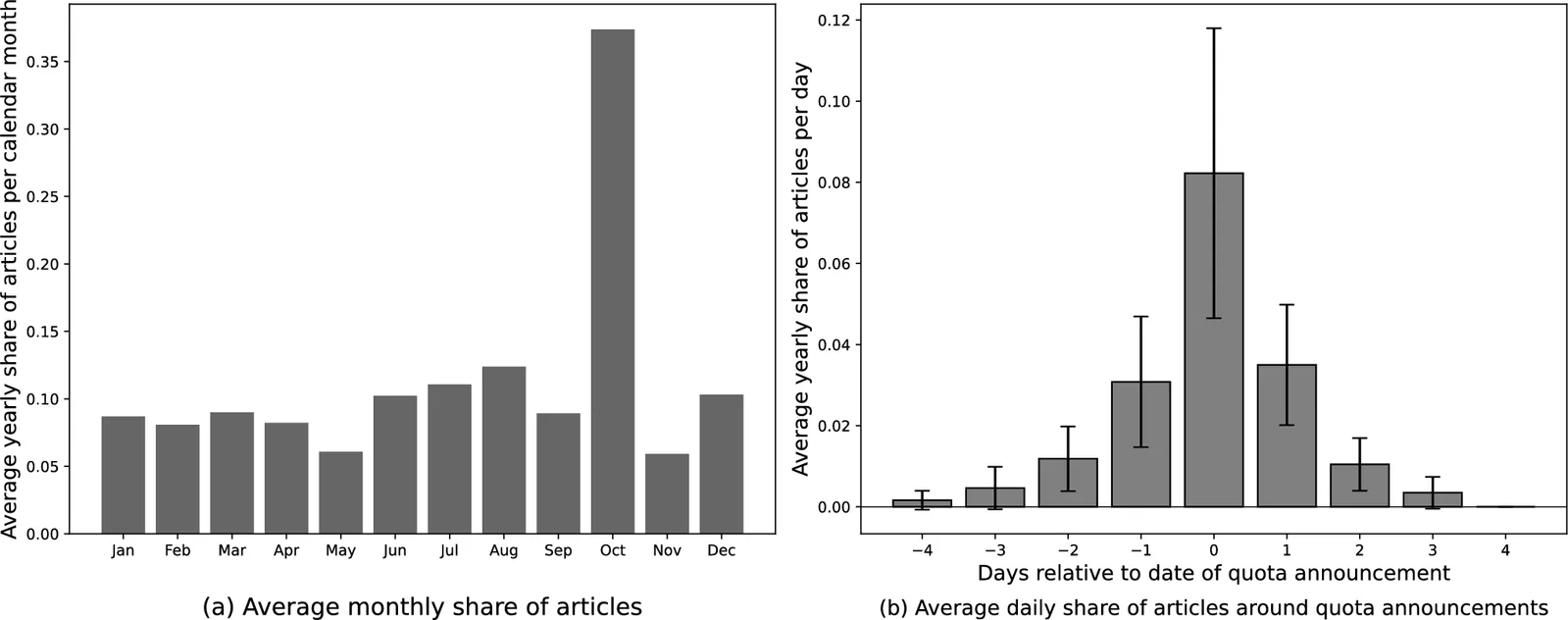
Frequency distribution of articles on the German Baltic fishery. **a** The bars show the average share of articles published in a calendar month. To account for the trend in published articles over our sample, we first computed the share of articles published in a calendar month of each of the years 2009–2021 and then took the sample average of all monthly shares. **b** The bars show the average share of articles around days of fishing quota announcements. We first computed the share of articles published in the period from four days before to four days after an announcement day for each of the years 2009–2021 and then took the sample average of all these daily shares. The grey intervals indicate 95% confidence bounds

The number of newspaper articles related to the German Baltic fishery is not constant across the year and rather follows a cyclical pattern. A salient peak in the number of published articles is observed in October, the month of the announcement of the EU Council’s decision about the fishing quotas for the upcoming year (Fig. 1a).

A daily inspection of October reveals that the peak is concentrated on just a few days around the fishing quota announcements by the EU Council (Fig. 1b). It starts two days before the announcement day and peaks on the day itself, when approximately 9% of all articles of a calendar year are published. In the subsequent two days, media attention is still elevated but gradually declines toward the usual level.

In contrast, the announcements of ICES advice for fishing quota in May are characterized by a tiny increase in media reporting to less than 1% of the articles per calendar year on the day of the announcement only, without any significant change in publication volume on the preceding or following days (see Fig. S5 in the Supporting Information).

The quantitative analysis also reveals that cod and herring are the two fish species that have by far the largest media coverage in our sample. On average, the share of newspaper articles mentioning cod and herring at least once is 47% and 52%, respectively, while plaice (16%), sprat (11%), and salmon (< 1%) follow with a large distance. While there is notable time variation, cod and herring lead in media attention in any calendar year in our sample (see Fig. S3 in the Supporting Information). Indeed, cod and herring dominate the public discourse on fisheries and fishery policies. This can be traced back to their ecological but particularly their socio-economic importance for fisheries and fishing communities today and in the past (Biester 1979; Döring et al. 2006, 2020; Schacht and Voss 2022). Even though the fishing quotas for both species have been strongly reduced in the past years, their importance in the media has not diminished. Therefore, in the following we focus on these two dominant species.

To capture the cyclical pattern of the news coverage documented above and relate newspaper sentiment to fishing quotas, we define a 'fishery year'. It starts one day after the announcement day of the fishing quotas by the EU Council and ends on the announcement day of the following year. For instance, the fishery year 2019 spans the period from the 16th of October 2019, one day after the announcement of 2019, until the 20th of October 2020, the announcement day of 2020. This approach ensures that our analysis accurately reflects the media *reaction* to fishing quota changes. Unless otherwise stated, the yearly data used in the subsequent analysis is based on the so-defined fishery years. Since our data extend until the end of the calendar year 2021, our last annual observation is the fishery year 2020, which ends in October 2021. Whenever we need a more detailed analysis, we disaggregate our 'fishery year' data to the quarterly frequency by defining the first quarter of each fishery year as the three-month period following the day of the fishing quota announcement, the second quarter as the subsequent three-month period and so on.

### Sentiment analysis of newspaper articles

Overall sentiment in the newspaper articles of our sample is consistently negative in all fishery years, with an average across all years of -0.25. This number means that the share of negative sentences exceeds the share of positive sentences by 25 percentage points, indicating a clearly negative tone throughout the sample. On average, only 4.4% of sentences show a positive sentiment, compared to 29.3% that are negative, while 66.3% of sentences are categorized as neutral. This result is consistent with the findings of Soroka (2006), Lengauer et al. (2012), and Soroka et al. (2018). It may support the notion of a negativity bias in media coverage, where a predisposition toward negative news can drive media outlets to emphasize adverse narratives. However, it may also reflect the exceptionally difficult situation of the German Baltic fishery (de Graaf et al. 2023; Lewin et al. 2023) including spatial conflict with numerous other stakeholder groups such as the offshore wind energy sector, resource conflicts with recreational fisheries but also natural predators such as grey seals (Barz et al. 2025), a sharp increase in various expenses such as fuel costs, and—partly as a consequence—a decline in the number of fishers and vessels, along with a lack of young people entering the profession. Future comparative studies will be necessary to determine which explanation is quantitatively more relevant.

**Fig. 2:**
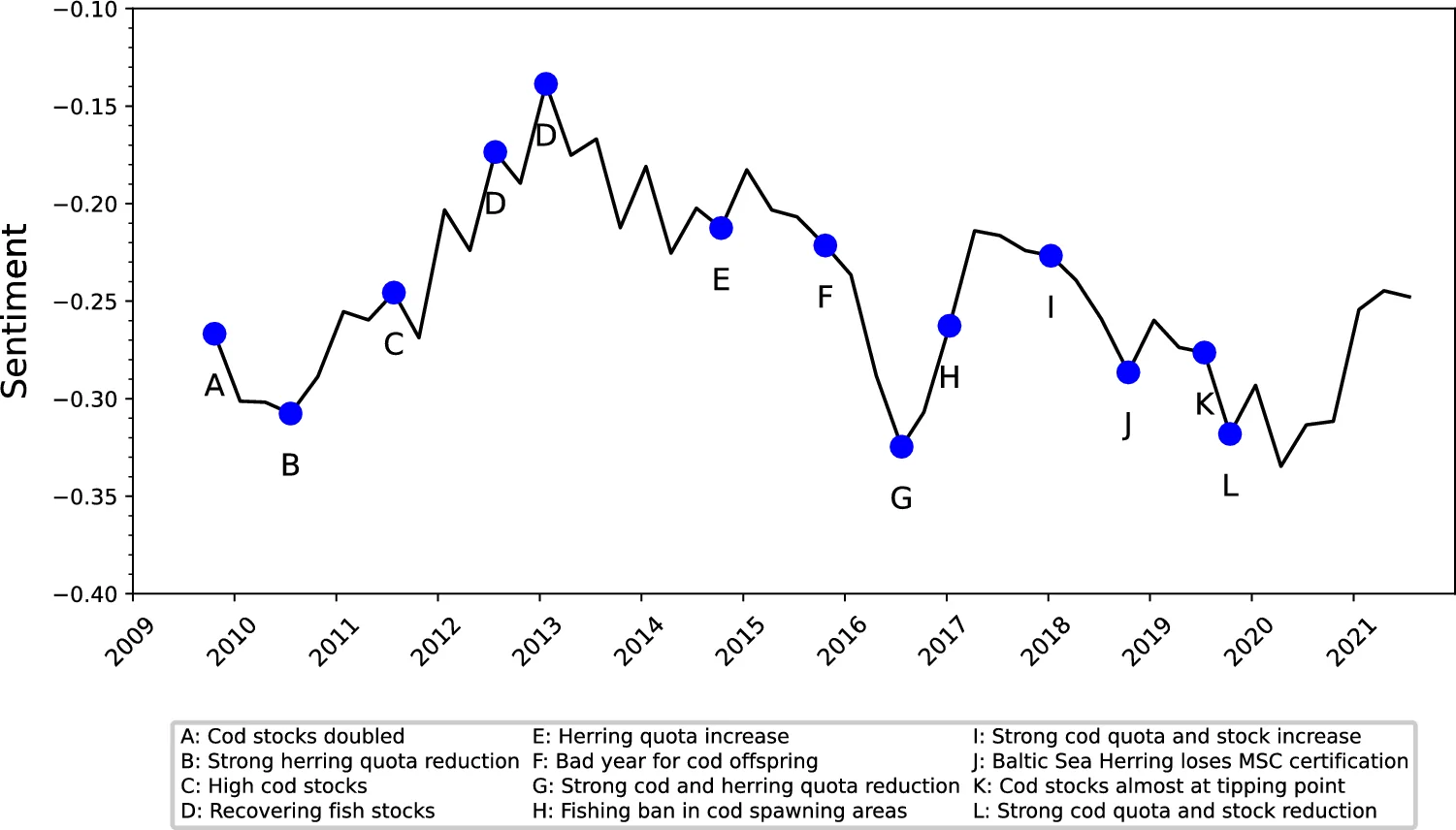
Sentiment and notable events. The solid line represents a 3-quarter rolling average of the quarterly sentiment index, which is calculated based on quarters defined by the ‘fishery year’. The blue dots represent important events, each marked with a corresponding letter

Around its average of − 0.25, newspaper sentiment exhibits considerable time variation, fluctuating between − 0.35 and − 0.15 (Fig. 2). These fluctuations are not random but linked to major events in the fisheries sector, which the newspapers reported. We marked these events with blue dots to highlight how strongly sentiment reacts, especially to changes in fish stock levels and fishing quota adjustments.

In years of substantial quota reductions, sentiment often takes a significant downturn, reflecting the immediate negative media reaction to these policy changes. For instance, the sharp declines in sentiment in 2016 and 2019 coincide with major quota reductions (Fig. 2, events G and L), illustrating the negativity bias of media sentiment to adverse regulatory decisions. Conversely, years with fishing quota increases, such as in 2018 (Fig. 2, event I), show an uplift in sentiment, indicating a more positive media outlook during periods of regulatory relief. Beyond fishing quota adjustments, other significant events like environmental certifications (Fig. 2, event J) and especially shifts in fish stock assessments (Fig. 2, events A, C, D, K) have also influenced media sentiment.

**Fig. 3:**
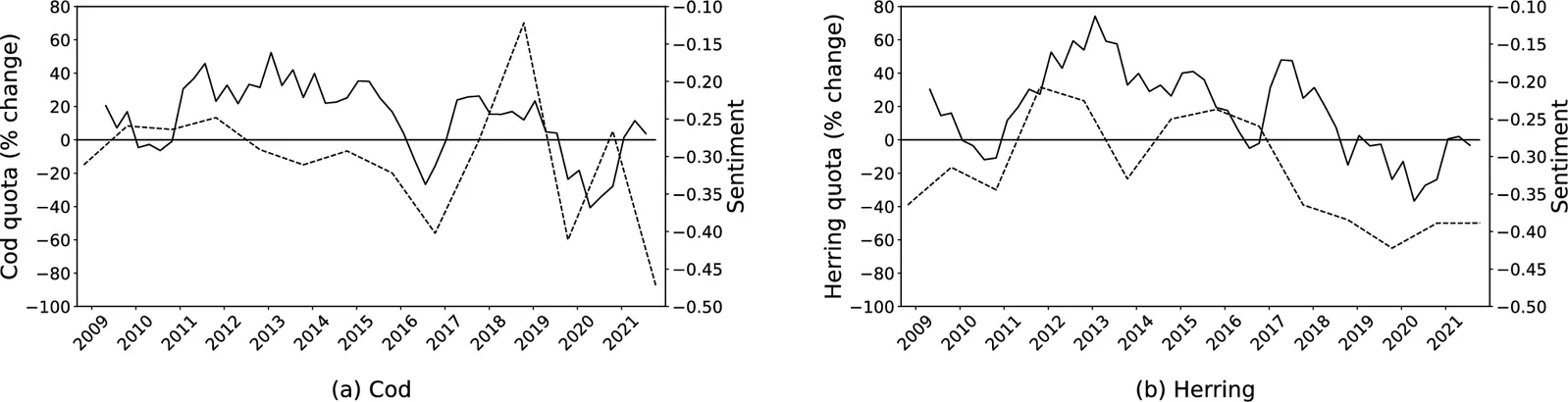
Sentiment and changes in fishing quota for cod (**a**) and herring (**b**). The solid lines represent the 3-quarter rolling average of quarterly sentiment indices, which are calculated for quarters defined by the ‘fishery year’. The dashed lines represent the percent changes in the respective German fishing quota compared to the previous year

A more detailed analysis of the link between fishing quota changes and sentiment needs to take into account that quotas are assigned species-specific. Therefore, Fig. 3 depicts fishing quota changes for the two most important species, cod (panel a) and herring (panel b), together with their respective sentiment at the frequency of a fishery quarter. It turns out that while the average quarterly sentiment indices for both fish species (-0.25 for cod and -0.24 for herring) are similar to each other and to the overall aggregate, their quarterly fluctuations differ significantly. To a large extent, they match the yearly fishing quota changes, which exhibit notable time variation with large peaks and troughs, even if fishing quotas have been on a declining trend in recent years.

For cod, the largest reductions in fishing quota were announced by the EU in 2016 (-56%) and 2019 (-60%), while the highest increase was negotiated in 2018 (+ 70%). Newspaper sentiment closely followed these changes, with strong declines in 2016 and 2019 and a temporary recovery in between. For herring, particularly negative sentiment values and fishing quota reductions coincide at the sample start, followed by more positive newspaper reports alongside rising fishing quota in the middle of the sample. The most recent years witnessed significant reductions in the fishing quota, with a minimum of -48% in 2019, which was matched by a very negative reporting tone in the same year.

Figure 3 also shows that quota changes are not the only determinant of newspaper sentiment. Deviations between the two time series are mostly related to other political events affecting the Baltic Sea fisheries. Important examples include the amendment of the CFP in 2013 (EU 2013), which was followed by a more positive reporting tone. Nevertheless, our analysis shows a noticeable relationship between the quarterly newspaper sentiment for cod and herring and their respective fishing quota changes. In particular, sharp cuts in the year-to-year fishing quotas are generally followed by a deterioration in sentiment.

### Analysis of stakeholders' media salience

We identified a large number of stakeholders, including 79 individuals and 37 organizations, to which the newspapers in our dataset refer at least 20 times. Organizations (number of observations: N = 5623) and individuals (N = 5534) appear roughly equally often, suggesting a balanced representation of persons and institutions. However, there are large differences between stakeholder groups. In particular, “eNGOs” are mentioned four times more often as organizations (N = 1758) than as individuals (N = 487), while “Fishery” stakeholders are typically referred to as individuals (N = 2510) and much less often as organizations (N = 862) (see Table S6a, b, c in the Supporting Information for a comprehensive stakeholder list).

Media salience, the number of yearly mentions of a group relative to the number of articles in that year, is roughly balanced between persons and institutions. Table 2 (panel a) shows the sample average of this measure by stakeholder group and institutional type (individual or organization) across all years of the sample. Counting stakeholders as often as they are mentioned within a single article, i.e., allowing for multiple counts per article, we find that an average newspaper article refers 6.72 times to stakeholders, of which 3.30 are individuals and 3.42 are organizations.

**Table 2:** Media salience and newspaper sentiment by stakeholder groups. Panel (a) shows the media salience of a stakeholder group calculated as the number of mentions for each group divided by the number of articles; panel (b) displays the newspaper sentiment computed as the difference between the shares of positive and negative group-specific sentences in the total number of group-specific sentences in each fishery year; panel (c) represents the fishery stance index calculated as the difference between the shares of the group-specific sentences articulating a socio-economic perspective and the share articulating an ecological perspective

	All groups	Politics & public authorities	Fishery	Science	eNGO
*(a) Media salience*					
Individuals	3.30	1.02	1.48	0.53	0.27
Organizations	3.42	1.25	0.57	0.52	1.09
Total	6.72	2.27	2.05	1.05	1.36
*(b) Newspaper sentiment*					
Individuals	− 0.32	− 0.28	− 0.31	− 0.37	− 0.47
Organizations	− 0.30	− 0.25	− 0.34	− 0.24	− 0.37
Total	− 0.31	− 0.26	− 0.32	− 0.31	− 0.38
*(c) Fishery stance index*					
Individuals	0.26	0.25	0.46	0.02	− 0.12
Organizations	0.08	0.13	0.59	− 0.08	− 0.16
Total	0.17	0.17	0.50	− 0.04	− 0.14

A breakdown by stakeholder group reveals that newspapers refer most often to “Politics & Public Authorities” (2.27 times per article), closely followed by “Fishery” (2.05), “eNGO” (1.36), and “Science” (1.05). This result deviates from previous studies, which found that political authorities are by far the most cited group in newspapers—for example, regarding the topics of animal protection and marine environmental risks (Jacobson et al. 2012; Jönsson 2011)—and thus are publicly given “the power to define problems and solutions” (Jönsson 2011). While political actors and public authorities are again the most frequently cited group in our sample, fishery representatives and fishers themselves rank closely behind.

A further implication of the stakeholder analysis is that the “Fishery” group is mentioned approximately 1.5 times as often as “eNGOs”. Hence, fishers’ voices are heard considerably more frequently than those of environmental protectionists. In addition, while individuals and organizations are represented more or less equally in the groups “Politics & Public Authorities” and “Science”, we note a strong imbalance of this distribution for “Fishery” and “eNGO”. “Fishery” is typically represented by an individual (1.48 out of 2.05 times), whereas “eNGO” is mostly mentioned as an organization (1.09 out of 1.36 times). These results remain largely intact when we count a stakeholder only once per article and thus neglect multiple counts of the same stakeholder within one article (see Tables S8, S9 and S10 in the Supporting Information).

### Sentiment of relevant stakeholder groups

After establishing that there is a difference between stakeholder groups concerning *who* is mentioned *how often* in newspaper articles, we next ask *how*, i.e., in which tone, they are mentioned. In panel (b) of Table 2, we report the sample means of newspaper sentiment by stakeholder group. Sentiment is not only negative on average as discussed above, but also negative for all stakeholder groups. Nevertheless, there are important differences between groups.

First, “Politics & Public Authorities” are mentioned in the least negative tone (− 0.28). A closer examination of quotations reveals the presentation of a “balancing” of political actors between environmental concerns and responsiveness to the concerns of local fishers. For example, one politician is quoted as saying: “We will hold talks with the federal government to find an agreeable solution”, and another quotation of a politician reads: “but the federal government will support the coastal fishers financially.” Overall, politicians seem to be presented as mediators engaged both in environmental and animal protection and in the economic future and well-being of fishers.

Second, newspapers refer to “eNGOs” in the most negative tone (− 0.38), while “Science” and “Fishery” are cited slightly more positively (− 0.31 and − 0.32). This difference mainly stems from a higher proportion of negative sentences associated with “eNGOs” compared to the other two groups. Analyzing the underlying quotations suggests that “eNGOs” typically act as warners and point out risks of insufficient marine protection measures. For instance, the increase in herring quota in 2011 was accompanied by stark warnings from the “eNGOs” concerning the potentially harmful outcome for herring stocks, while the decrease in cod quota in 2016 was criticized by “eNGOs” as both too late and not substantial enough to help the already deteriorating stocks.

Distinguishing between individuals and organizations of the respective stakeholder group yields additional insights. While overall sentiment is almost identical between individuals and organizations (− 0.32 vs. − 0.30), there are large differences in the “Science” group and especially in the “eNGO” group, where sentences mentioning individuals are much more negative (− 0.47) than sentences referring to organizations (− 0.37). In contrast, individuals of the “Fishery” group are even framed slightly more positively (− 0.31) than their organizations (− 0.34). We conclude that there is an important gap in the newspaper tone when it comes to reporting on individual fishers and their spokespersons as opposed to representatives of “eNGOs” (and also”Science”).

**Fig. 4:**
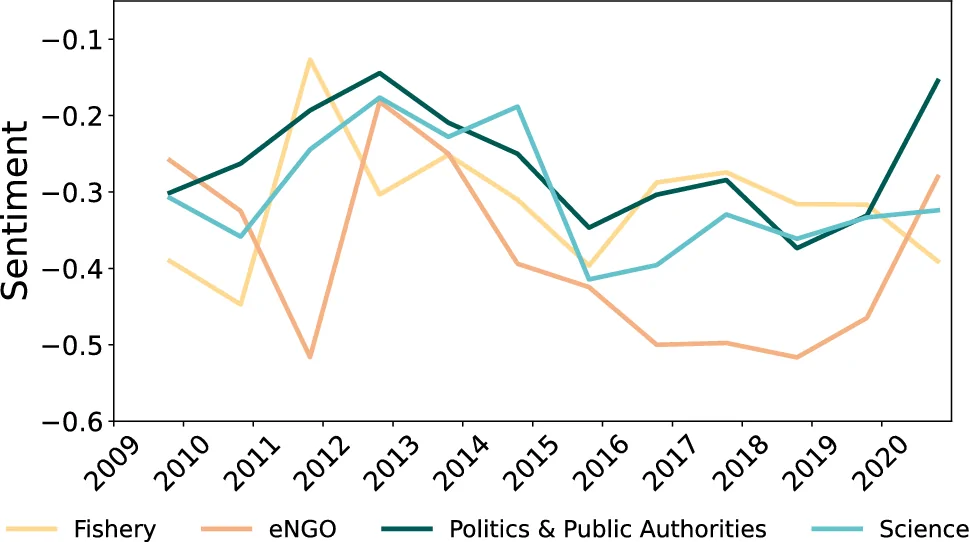
Yearly sentiment index for each stakeholder group. The figure shows stakeholder-specific newspaper sentiment for the fishery years 2009–2020 illustrated in different colors (petrol:”Politics & Public Authorities”, yellow:”Fishery”, turquoise:”Science”, orange:”eNGO”)

We finally analyze the evolution of group-specific newspaper sentiment in our sample (Fig. 4). A first result is that—while the aforementioned level differences persist over time—the overall sentiment trend is generally similar across groups, with “Politics & Public Authorities” and “Science” evolving particularly closely. Nevertheless, the sentiments for the “Fishery” and “eNGO” groups show pronounced temporary deviations from this trend.

One such episode occurred in the fishery years 2010–2011, when the Fishery sentiment jumped upwards from an all-time low of − 0.45 to its sample maximum of − 0.13, while the “eNGO” sentiment moved in the opposite direction from − 0.33 to − 0.52. In the fishery year 2011, “eNGO” stakeholders were particularly disappointed that the fishing quotas for herring and cod were not reduced despite earlier studies warning of a collapse of fish stocks in the Baltic Sea, while fishers were relieved that the quotas were higher than previously feared. What had happened? The herring quota had been drastically reduced in the fishery years 2009 and 2010, putting a large economic burden on the German Baltic fishery and thus contributing to the very negative “Fishery” sentiment in 2010. In 2011, the EU Council increased the herring quota by 30%. This corresponded with a sharp improvement in the “Fishery” sentiment as newspapers also reported very high herring catches with low fishing effort, coupled with high prices for this target fish species. Why was the reporting tone with respect to “Fishery” nevertheless negative? According to the newspaper reports of 2011, a number of challenges remained, such as high fuel prices, high levels of bureaucracy, and the associated decline in the number of fishers who blamed the EU fisheries policy for this development.

The reporting tone with respect to “eNGO” moved in the opposite direction and declined sharply. Unsurprisingly, in newspaper articles of the fishery year 2011, “eNGO” emphasized the precarious state of the Baltic Sea that was expected to further deteriorate after the quota increase. Specifically, they referred to the poor situation of the harbor porpoise, high levels of nutrient and pollutant inputs, intensive fishing, increasing shipping traffic, and gillnet fisheries, all of which put a heavy burden on marine organisms and habitats.

Given that the 2011 herring quota increase spurred opposite reactions of the “Fishery” and “eNGO” sentiments, why do we also see movements in the same direction? An insightful example is the fishery year 2015, when the EU Council announced a strong reduction in the cod quota. It was followed by a sharp decrease in the reporting tone with respect to both “Fishery” and “eNGO” (and also to “Science” and “Politics & Public Authorities”)—but for very different reasons as the newspaper articles of that year indicate. While the quota is still characterized as too high by “eNGO” due to the poor ecological condition of cod, fishers and their spokespersons see their individual livelihoods, and the fishing industry more generally, threatened due to income losses. Hence, newspaper reporting is negative for both groups, even if one group argues on an ecological level and the other group on a socio-economic level.

### Fishery Stance: stakeholders’ views on the Baltic Sea fishery

How do the media portray the stakeholders’ views on the Baltic Sea fishery? In panel (c) of Table 2, we report the sample means of the newspaper fishery stance index by stakeholder group. The average fishery stance index across all groups is slightly positive (0.17), indicating that there are, on average, 17 percentage points more sentences articulating a socio-economic stance than an ecological one. “Politics & Public Authorities” exhibit the same stance of 0.17, corroborating their overall balanced role already discussed above. By contrast, “Science” (− 0.04) and especially “eNGOs” (− 0.14) are portrayed with a stance that prioritizes ecological concerns, while “Fishery” (0.50), not surprisingly, is portrayed with the stance most strongly focused on socio-economic needs (see Table S11 in the Supporting Information).

A breakdown by individuals versus organizations does not yield any additional strong conclusions. Nevertheless, it suggests that “Politics & Public Authorities” is not a homogeneous group: organizations (i.e., mostly public authorities) are relatively neutral (0.13), while individuals (i.e., mostly politicians) exhibit a much stronger socio-economic stance with an index value of 0.25. To shed more light on this heterogeneity, we further split the group “Politics & Public Authorities” into the traditionally most relevant German political parties – the Social Democratic Party (SPD), the Christian Democratic Union (CDU), and the Green Party – and all other entities, which consist mostly of public authorities.

While the SPD is a center-left party traditionally associated with labor interests and economic support for rural and working-class communities, the CDU is a center-right party with a strong base in rural areas and among conservative voters. The Green Party is a progressive party with a strong environmental political agenda.

It turns out that politicians of the SPD are portrayed as having a strong socio-economic perspective (index value 0.42), followed closely by the CDU (0.39), while the Greens are framed as almost neutral (0.04) (see Table S12 in the Supporting Information).

Importantly, this newspaper framing reflects the positions of these parties as outlined in their official election manifestos for the two German federal states bordering the Baltic Sea, Schleswig–Holstein and Mecklenburg-Vorpommern. According to these documents, the Green Party is the most critical of the fisheries sector, emphasizing the ecological damage caused by overfishing, the need for enhanced environmental protection, and stricter regulations on the fisheries. In contrast, the CDU strongly underscores the cultural and economic importance of fisheries, advocating for support measures such as higher quotas and financial aid. Similarly, the SPD emphasizes the importance of maintaining a sustainable fishery sector, with a strong focus on its economic viability and the security of jobs in coastal communities.

### Thematic priorities of relevant stakeholder groups

**Fig. 5:**
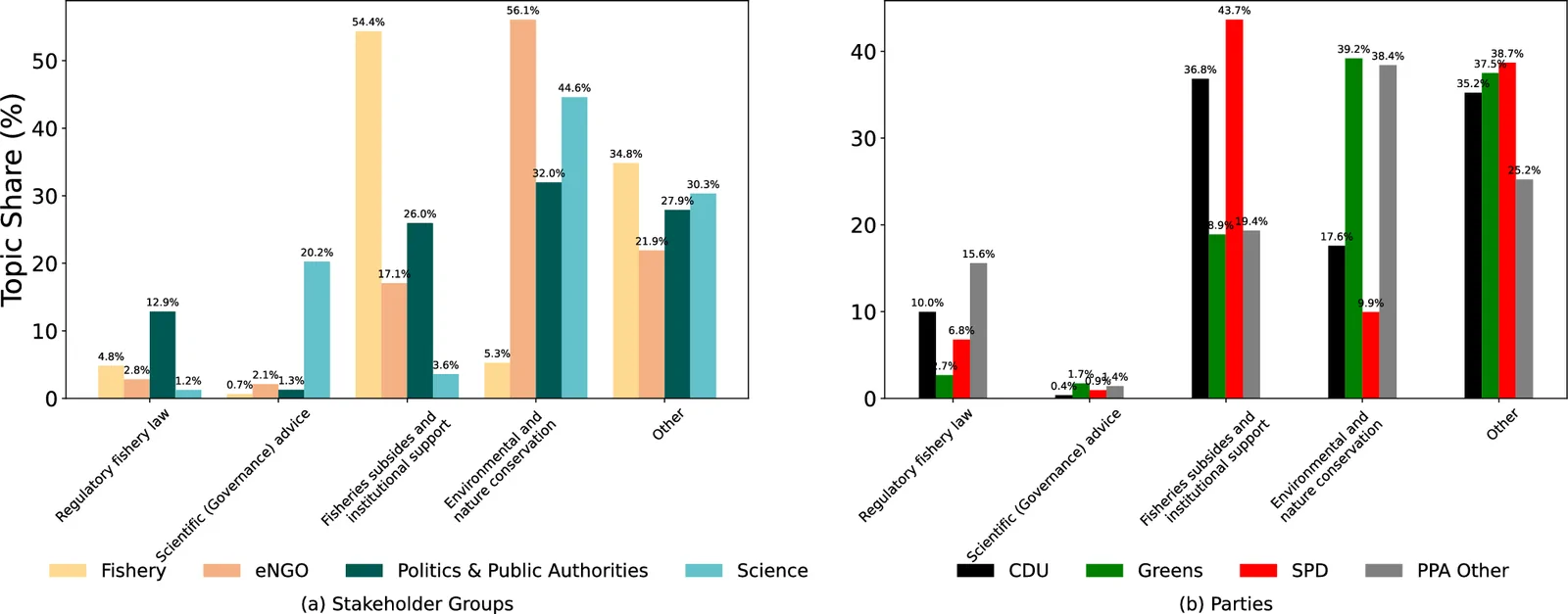
Topic shares for each stakeholder group (**a**) and party (**b**). **a** shows stakeholder-specific newspaper topic shares over the full sample from 2009 until 2020 illustrated in different colors (petrol:”Politics & Public Authorities”, yellow:”Fishery”, turquoise:”Science”, orange:”eNGO”). **b** represents party-specific newspaper topic shares over the full sample from 2009 until 2020 illustrated in different colors (black: CDU, green: Greens, red: SPD, grey: PPA Other, i.e., all “Politics & Public Authorities” stakeholders excluding the three parties

We finally analyze the fishery-related topics with which individual stakeholder groups are most concerned. This analysis reveals that the discourse is divided along two main lines (Fig. 5a, b). On one side, sentences mentioning “Science” and “eNGO” predominantly focus on environmental concerns: 44.6% of the sentences related to “Science” and 56.1% of those related to “eNGO” address sustainability issues and environmental protection (Fig. 5a). On the other side, sentences from the “Fishery” group more frequently focus on fishery topics and the support needs of the fisheries sector, with 54.4% of fishery-related sentences covering these issues. “Politics & Public Authorities” again assume an intermediary role, with 26.0% of sentences addressing fishery issues and 32.0% focusing on environmental concerns.

The remaining topics, regulatory fishery law and scientific (governance) advice, each account for only a small share across most groups. “Politics & Public Authorities” mention regulatory law in 12.9% of their coverage, reflecting their role in setting and enforcing legal frameworks, while “Fishery” (4.8%), “eNGO” (2.8%), and “Science” (1.2%) discuss this topic only occasionally. By contrast, scientific advice appears in 20.2% of sentences from “Science”, yet remains below 3% for “Fishery” (0.7%), “eNGO” (2.1%), and “Politics & Public Authorities” (1.3%).

A breakdown of the “Politics & Public Authorities” group into the three relevant political parties and all other members again reveals pronounced heterogeneity (Fig. 5b). The media cite the Social Democratic Party (SPD) and, with some distance, the Christian Democratic Union (CDU) typically in the context of aid and support for the German Baltic fishery, while they mention the Greens when the topic of sustainability and environmental protection is discussed. Interestingly, the same holds for the other group members, which consist mostly of public authorities: they are predominantly cited in the context of environmental protection (Fig. 5a).

## Discussion

Systematically analyzing around 1800 newspaper articles, this study provides new insights into how German print media portray Baltic fishery issues. We summarize these insights as four key results.

First, using a quantitative analysis of newspaper coverage across the year, we found that the German media discourse focuses on cod and herring, the two species traditionally most relevant, and that the impact of political decisions acts as a critical catalyst of the frequency of reporting. The yearly announcements of the new fishing quota by the EU Council, representing decisions with immediate economic consequences, crucially increase the newspapers’ thematization of fishery-related topics. In contrast, the ICES advice only has a marginal effect on the media discourse. This politicization effect is very short-lived, as the number of newspaper articles decreases sharply just a few days after the announcement.

A detailed stakeholder analysis yielded our second key result, which has three dimensions: the fishery group is mentioned considerably more often than the eNGO group; fishers and fisheries representatives are typically mentioned as individuals, whereas eNGOs are portrayed primarily as organizations; when individuals of the eNGO group are cited, it is often in a particularly negative tone. These patterns imply that fishers and fisheries representatives have a twofold advantage over eNGOs in the public discourse: They are heard more often, and their cause is presented in a more positive context and individualized way, whereas eNGOs rather appear as “faceless” organizations or as pessimistic warners. Scientists are mentioned in a balanced manner—almost equally as individuals and as organizations—but when quoted, their tone is predominantly negative. Overall, the results suggest that the newspapers portray the worries of the fishers and fisheries representatives in a more personal and individualized style than environmental protection concerns.

Our third key result is a strong positive correlation between media sentiment and changes in herring and cod fishing quotas: an announced reduction of fishing quotas is typically followed by a decline in media sentiment. This suggests that the immediate adverse consequences for the fishery sector receive a higher weight in the media coverage than the benefits for the marine ecosystem (which may contribute to the recovery of cod and herring stocks in future years). A more in-depth analysis of two episodes of quota changes offered an additional explanation. The negative response is partly due to the better representation of fishers’ concerns in the media, but it partly also reflects that not only fishers complain about quota reductions but also eNGOs. While the former criticize the economic hardships of quota reductions, eNGOs assess them as insufficient from an ecological point of view. This reinforces the interpretation of the role as pessimistic warners prescribed by the media to eNGOs.

Our fourth key result is that there are huge differences between stakeholder groups regarding their position on the German Baltic fisheries. On one side, the fishers (including their representatives) and two political parties, the SPD and the CDU, are portrayed with a stance that strongly prioritizes the socio-economic needs of the industry, a focus that is also reflected in their frequent references to fishery aid. On the other side, scientists, eNGOs, and the Green Party are, on average, more strongly concerned about fishery-related environmental and sustainability issues. These camps can be seen as representing two distinct advocacy coalitions in the German media debate, one focused on the industry's viability and the other on the ecosystem's health (see also Nilsson and Sandström 2024).

While a systematic discursive evaluation of fisheries-related quotations is beyond the scope of this paper, in the following, we provide an illustrative overview of how fisheries are portrayed. Broadly, we find that the newspapers in our sample emphasize the concerns of both the German Baltic fishery sector as a whole and at the individual level. For instance, a quote from the general manager of the fisheries association of the federal state of Schleswig–Holstein reads: "With this uncertain situation, how are we to attract young people to the fishing profession?”.^3^ Another quote from him in a different newspaper is: “Not only catch quotas and rigid EU bureaucracy made life difficult for them”.^4^ At the individual level, newspapers provide narratives about the daily experiences and practical, personalized challenges faced by fishers. One text passage reads: “As a full-time fisher, Matthias Nanz also has to cast his nets in the harsh winter. But it's not just the cold temperatures that cause him problems, it is also cormorants and EU quotas.”^5^ Other articles highlight the strong affectional attachment of fishers to their profession, thereby probably contributing to the not-so-negative newspaper sentiment of individual fishers compared to eNGO representatives, e.g., “Detlefsen loves his job and can't imagine doing anything else.”^6^

Taking into consideration the high frequency of fisheries citations as well as the illustrated discursive practices of personalization and individualization of delineated concerns, the analyzed newspapers, especially the regional ones, can be interpreted as a kind of “advocates” of the fishers and their representatives providing them some agenda-setting voice for influencing the media discourse according to their concerns and interests by means of promoting identification with, and empathy for, specific protagonists (Glaser et al. 2009; Fernandez-Quintanilla 2020). The media framing of fishers in the German Baltic Sea context thus seems to be more positive compared to the much more ambivalent media or even negative representation of the aquaculture and agriculture sectors documented by previous research. In line with this interpretation is also the finding that eNGOs and scientists, who are more critical of the fishery sector, are less frequently given the public media floor for the topic than fishers and fisheries representatives.

Our results, moreover, have shown that regarding eNGOs, we observe a large number of organizations mentioned and markedly fewer individuals. This stands in sharp contrast to the discourse of personalized concerns of fishers, and may be seen as a media practice to underline the authority of eNGOs as rather objective and neutral experts. In the following, we provide some illustrative examples, which should be more systematically analyzed in future research. For example, within the newspaper *Die Welt*, we can read: “as the WWF informed on Friday in Hamburg, the stock in the Eastern Baltic Sea between Bornholm and Finland has already dropped by a third.”^7^

## Conclusion

To summarize, we find that the media coverage of the German Baltic fishery is characterized by clear differences in the frequency of naming specific actors/groups, the sentiments associated with them, and the group-specific narratives. While our dataset does not allow us to analyze the empirical effect on readers’ perceptions and possibly on their (voting) behavior, media influence should not be underestimated, especially in areas that are not part of the readers’ daily lives (Jacobson et al. 2012) which is certainly the case in the German Baltic fishery discourse.

Our analysis provides an evidence base contributing to the development of more effective communication and management strategies. The consistent patterns we identified in German media coverage suggest several concrete paths forward for stakeholders. For example, our finding that media attention is catalyzed by political decisions rather than scientific advice highlights a critical communication gap. To ensure their findings inform public debate, scientific bodies like ICES should adopt a proactive media strategy, for example, by using targeted press releases and accessible summaries to explain the ecological rationale for their advice well ahead of the political negotiations. Similarly, the stark imbalance in how stakeholders are portrayed offers strategic insights. While fishers' concerns are personalized, eNGOs are often presented as abstract, negative organizations; to counter this, eNGOs could shift from organizational warnings toward more personalized narratives that tell the stories of individuals affected by ecosystem change or highlight successful conservation efforts. This challenge of media negativity also extends to policymakers, who face this bias when implementing necessary quota reductions. A robust communication strategy that is deployed simultaneously with any cuts, framing them as a long-term investment in the environment and in socio-economic well-being and coupling them with concrete support measures, is therefore essential. More broadly, our finding that the media portrays two opposing "coalitions" can itself be used as a strategic tool. Stakeholders interested in de-escalating conflict can use this "map" of the media landscape to design more effective dialogue forums, focusing on shared goals to move beyond the conflict-driven narratives dominant in the media. By understanding these media logics, stakeholders can transition from being passive subjects of the news cycle to becoming active participants in shaping a more balanced public discourse.

One should note, however, that our results are based on (online) newspaper articles. This matters because media consumption differs across age groups and across information repertoires. For Germany, Wunderlich and Hölig (2022) show that social media is reported as a daily source of information much more often among younger respondents than among older respondents, whereas (printed) newspaper use increases strongly with age. Accordingly, additional work would be needed to assess whether similar dynamics hold in other channels, especially social media.

While the model we developed focuses explicitly on the German Baltic fishery, it provides a tool that can be easily adapted to other topics and case studies whenever corresponding datasets of newspapers or related media are available.

Further research could identify the drivers behind these patterns in coverage by conducting qualitative interviews or focus groups with eNGOs, fishers, and journalists to unpack communication strategies and constraints; compare coverage in other Baltic or EU fisheries; link media frames to audience perceptions and policy legitimacy using surveys or experiments; and widen the corpus beyond newspapers to social media, television, and radio to see how different media platforms shape agenda-setting. This study focused exclusively on the German newspaper landscape, which has its own market dynamics, journalistic culture, and set of regional and national publications. Consequently, the specific patterns of stakeholder representation and sentiment we identified may not be directly generalizable to other countries. Future comparative research could explore how the discourse on fisheries differs across the various national media systems of the Baltic states, thereby providing a more holistic understanding of the public debate.

Lastly, our study and applied methodological approach are exclusively of quantitative nature. However, the collected dataset offers multiple opportunities to conduct a more comprehensive qualitative content analysis. For instance, a qualitative analysis could examine the specific language, such as terms of conflict, that explain why political quota announcements consistently overshadow scientific advice. Furthermore, it could deconstruct the narrative strategies used to construct fishers as personalized protagonists while framing eNGOs as abstract, pessimistic organizations. This might provide a deeper insight into different system understandings and problem framing related to the German Baltic fishery, thus creating a valuable complement to our findings.

## Supplementary Information

Below is the link to the electronic supplementary material.

Supplementary file1 (PDF 1141 MB)

## Data Availability

The data presented in this study are available on request from the corresponding author. The data are not publicly available due to restrictions, i.e. privacy and ethics. The programming code for this paper is available through https://github.com/Jasper-Baer/Media-s-Role-in-Agenda-Setting-Exploring-Media-Coverage-of-the-German-Baltic-Fishery-Discourse.

## Footnotes

1 https://www.lexisnexis.de/.

2 All four annotators are fluent in German.

3 From Spiegel (2011). Reduzierter Fischfang. EU-Länder kritisieren geplanten Fangquoten “. https://www.spiegel.de/wissenschaft/natur/reduzierter-fischfang-eu-laender-kritisieren-geplante-fangquoten-a-771241.html.

4 From Hamburger Abendblatt (2010). “Fischer fühlen sich gefangen”.https://www.abendblatt.de/region/norddeutschland/article107815538/Fischer-fuehlen-sich-gefangen.html .

5 From SHZ (2009).“Erschwerte Bedingungen für Holmer Fischer”. https://www.shz.de/lokales/schleswiger-nachrichten/erschwerte-bedingungen-fuer-holmer-fischer-id918726.html.

6 From SHZ (2009). “Jagd auf den Quotendorsch in unbekannten Gewässern”. https://www.shz.de/lokales/schleswiger-nachrichten/jagd-auf-den-quotendorsch-in-unbekannten-gewaessern-id982516.html.

7 From Die Welt (2006). “WWF-Studie: Der Ostsee-Dorsch ist akut gefährdet”. https://www.welt.de/print-welt/article88868/WWF-Studie-Der-Ostsee-Dorsch-ist-akut-gefaehrdet.html.

## Declarations

### Supporting information

Additional results may be found online in the Supporting Information section at the end of this article.
