# Supplementary material for: Media’s role in agenda-setting: Exploring media coverage of the German Baltic fishery discourse
Source: Ambio. 2026 Mar 25;55(10):2498–515. doi: 10.1007/s13280-026-02371-5 (PMC13553996; doi:10.1007/s13280-026-02371-5)
Supplement: Supplementary file 1 [file 13280_2026_2371_MOESM1_ESM.pdf]

Ambio

Supporting Information

This supporting information has not been peer reviewed.

Media's Role in Agenda-Setting:

Exploring Media Coverage of the German Baltic Fishery Discourse

This Supporting Information includes:

1. Supporting Material and Methods
2. Supporting Summary Statistics
3. Supporting Results
4. Supporting References

1. Supporting material and methods

1.1. Text Classification with BERT

To classify the sentiment of each sentence in our dataset, we use BERT (Bidirectional Encoder Representations from Transformers) proposed by Devlin et al. (2019). BERT is a language model that represents words in a sentence by modeling how each word relates to all other words in that sentence. This context sensitivity is important for sentiment classification, for example to handle negation such as “not good”.

For sentence-level classification, BERT produces a vector representation of the full sentence. We feed this vector into a simple classification layer. The classifier outputs class probabilities (e.g., positive, neutral, negative) and assigns the sentence to the class with the highest probability.

BERT is an encoder-only Transformer model, based on the Transformer architecture introduced by Vaswani et al. (2017). Depending on the variant, it consists of a stack of encoder layers (for example, 12 or 24 layers). Each encoder layer has two key components: multi-head self-attention and a position-wise feed-forward network.

Self-attention is the mechanism that lets the model use the full sentence context when updating each word representation. For a given word (more precisely, token), the model computes how strongly it should attend to every other token in the sentence. It then updates the token representation by taking a weighted combination of information from the other tokens. Multi-head attention repeats this process in parallel in several ``heads'', so different heads can focus on different types of relationships, such as negation, modifiers, or longer-range dependencies.

After the self-attention step, the feed-forward network applies a learned non-linear transformation to each token representation. Its role is to re-express the information that was

gathered by attention in a richer feature space, making it easier for the next layer to work with and, ultimately, for the classifier to separate sentiment classes.
After many attention and feed-forward updates, the model produces representations that can be used by a simple classifier. These representations are not learned from the sentiment task alone. Instead, BERT is first pre-trained on a general language objective and only then fine-tuned on the labeled sentiment data. The pre-training process consists of two steps.

The first step is the ``Masked Language Model'' (MLM) task. First, all words in a given sentence are split into a set of tokens. Each token corresponds to a word or a part of a word in sentence. For a detailed description of the tokenization process, see Wu (2016). Additionally, a classification token is added at the beginning of each sentence. This token signals that the ensuing tokens will be used for classification purposes in subsequent steps of the model. Next, 15\% of all words in a sentence are replaced with a masking token. This masking token acts as a placeholder to hide the original word from the model during training. The model then predicts the original words based on the remaining words in the sentence. This allows the model to learn the meaning of a word based on the surrounding context.

The second task is called ``Next Sentence Prediction'' (NSP). The model receives a pair of sentences and has to predict if the second sentence in the pair is the subsequent sentence in the original document. During the training process, 50\% of the second sentences in the pairs are subsequent sentences, while 50\% are random sentences from the rest of the dataset. This step trains the model to learn the context across sentences.

Due to the small size of our dataset and limited available hardware, instead of training our own model from scratch, we used a pre-trained model from Chan et al. (2020) which was trained on a large German dataset.

To adapt this pre-trained model to our specific task of sentiment, fishery stance and topic classification, we fine-tuned it on our annotated training datasets. This involves adding a fully connected layer on top of the Transformer output for the classification token. This layer generates a probability distribution across the specified classes. For instance, for our

sentiment analysis, we have three classes—positive, neutral, and negative—reflecting the sentiment of the sentence. The classification of the sentence is determined by the class receiving the highest probability score in this output distribution. The full model, using the weights from the pre-training phase as initial weights, including the added classification layer, was then fine-tuned on our annotated training dataset to obtain our final models.

After training our models, we classified all sentences in our dataset. Based on the classified sentences we created quarterly and yearly indices for sentiment and fishery stance. For the sentiment index, we computed

$$sent_t = \frac{s_{pos}^t - s_{neg}^t}{s_{pos}^t + s_{neu}^t + s_{neg}^t}$$

where $sent_t$ is the sentiment in period *t*, s_{pos}^t is the number of positive sentences in period *t*, s_{neg}^t is the number of negative sentences in period *t*, and s_{neu}^t is the number of neutral sentences in period *t*. Hence, a negative value indicates that there are more negative than positive sentences in the corresponding period. Similarly, for the fishery stance we computed

$$stance_t = \frac{f_{socio}^t - f_{eco}^t}{f_{socio}^t + f_{neu}^t + f_{eco}^t}$$

where $stance_t$is the fishery stance in period *t*, f_{socio}^t is the number of sentences focused on socio-economic viability in period *t*, f_{eco}^t is the number of sentences focused on ecological health in period *t*, and f_{neu}^t is the number of neutral sentences in period *t*.

1.2. Creation of Training Datasets

To ensure consistent annotations, we developed annotation guidelines that define the classes for each task. For the sentiment analysis training dataset, the guidelines delineate definitions for positive, neutral, and negative sentences. We randomly drew 4000 sentences from our

dataset which were classified according to their sentiment by four annotators using the annotation guideline.

For our fishery stance training dataset, we randomly selected 2500 sentences. These sentences were annotated according to a guideline specifically designed for fishery stance, categorizing each sentence into one of three classes: (i.) Focused on Socio-Economic Viability: Sentences that emphasize the economic, cultural, or operational needs and challenges of the fishery sector. These statements often highlight the importance of fishing for communities and advocate for measures that support the industry's viability. (ii.) Focused on Ecological Health: Sentences that highlight environmental concerns, risks to fish stocks, or broader ecosystem sustainability. These statements often advocate for precautionary measures, stricter regulations, or conservation efforts to protect the marine environment. (iii.) Neutral: Sentences that present factual or descriptive information without any clear judgment.

For the topic analysis training dataset, 3000 sentences were annotated into five categories:

Regulatory fishery law, Scientific (governance) advice, Fisheries subsidies and institutional support, Environmental and nature conservation, and Others. Regulatory fishery law focuses on stock management issues, including quota allocation, fishing restrictions, and other regulatory measures governing fisheries. Scientific (governance) advice related sentences include scientific advice for fishery governance, such as stock assessments. Fisheries subsidies and institutional support encompass both the various forms of financial, technical, and institutional assistance provided to fishers and the specific needs and perspectives of the fishers themselves. Sustainability and environmental conservation sentences cover topics related to ecological concerns and efforts aimed at safeguarding the environment. Finally, the Others category includes sentences that do not clearly fit into the previous categories.
Each sentence in our training datasets was labeled by three annotators at a time, and after the annotation process a majority vote was applied to determine the final label for each sentence. The sentiment analysis dataset was annotated by the first and second authors along with two instructed student assistants, whereas for the fishery stance and topic analysis datasets the

annotations were conducted by the first author and two instructed student assistants. To ensure consistency, we developed annotation guidelines that all annotators followed during their annotation process.

1.3. Annotation Guidelines

The following annotation guidelines were developed to ensure consistency and clarity in the classification process. They define the criteria for each classification task, and provide detailed instructions for annotators.

1.3.1. Sentiment Annotation Guideline

Objective and Subjective Sentences

Initially, it is to be determined by the annotators whether a sentence is objective or subjective. A sentence is classified as objective if provided by the author or the cited person, it presents a purely descriptive statement devoid of any judgment or emotion, relying solely on factual (though not necessarily accurate) information. Such sentences are consistently to be annotated as neutral. Even if parts of the information in the sentences might be interpreted as positive or negative from various viewpoints, the annotation of the sentence is to remain neutral. This particularly applies to sentences that announce changes in fishing quotas. For instance, the sentence “Cod quotas are reduced for next year” is to be annotated as objective, thereby considered neutral. These sentences are to be marked with a “1” (excluding the quotation marks). Instances of these types of sentences from the corpus are exhibited in table S1.

Table S1: Objective sentences. The table shows examples of objective sentences in the original (German language, left column) and in translation (English language, right column).

Sentence	Translation
Männliche Kegelrobben können bis zu 300 Kilogramm wiegen.	Male grey seals can weigh up to 300 kilograms.
Vor drei Jahren wurden 320 Naturfelsen versenkt.	Three years ago 320 natural rocks were sunk.
In der EU sollen neue Fangquoten für die Ostsee gelten.	In the EU, new fishing quotas are to apply to the Baltic Sea.

A sentence is identified as subjective when an opinion or emotion towards an entity is expressed by the author or the person being cited. The sentiment may be conveyed directly by the author, or through direct or indirect speech of another person or organization. For instance, in the sentence “The politician praises the new law he helped to pass,” the sentiment (positive) attributed to the politician is to be taken into account during annotation. Subjectivity can sometimes be directly signaled through words such as “good,” “bad,” “concerning,” etc., which clarifies the annotation process. Nonetheless, rhetorical questions, metaphors, irony, or other rhetorical devices are often employed by authors to convey their opinions indirectly.

When a sentence is determined to be subjective, the subsequent task involves deciding whether its sentiment is positive or negative. Sentences with positive sentiments are to be marked with a “2,” and those with negative sentiments with a “0” (excluding the quotation marks). The intensity of the sentiment is not to be considered. Both “Today was a nice day” and “Today was a wonderful day” are to be annotated simply as positive. Examples of subjective sentences from the corpus are presented in table S2.

Table S2: Subjective sentences. The table presents examples of subjective sentences in the original (German language, left column), in translation (English language, middle column), and the chosen sentiment (right column).

Sentence	Translation	Sentiment
”Dem Hering in der westlichen Ostsee geht es miserabel,” sagt Christopher Zimmermann, Leiter des Thünen-Instituts für Ostseefischerei.	”The herring in the western Baltic Sea is doing miserably”, says Christopher Zimmermann , head of the Thünen Institute for Baltic Sea Fisheries.	negative
Fischer wie Randy Repenning fürchten um ihre Existenz.	Fishermen like Randy Repenning fear for their existence.	negative
Zufrieden sind weder Fischer noch Umweltschützer.	Neither fishermen nor environmentalists are satisfied.	negative
Auch die Wissenschaft irrt mitunter.	Even science is sometimes mistaken.	negative
Eingespielte Routine, eine feste Konstante seit immerhin 55 Jahren.	Well-established routine, a constant for 55 years now.	positive
”Die Entscheidung werten wir als kleinen Erfolg”, sagte der Geschäftsführer des Landesan-glerverbandes Axel Pipping.	”We consider the decision a small success”, said the managing director of the State Anglers Association Axel Pipping.	positive
Positiv schätzte der Verband die Entwicklung der Populationen in Nord- und Ostsee ein.	The association assessed the development of the populations in the North Sea and Baltic Sea positively.	positive

Special Cases

Neutral reporting: Reporters tend to have a neutral writing style and rarely express their own sentiment directly. In some cases, this can lead to sentences that are objective but would be interpreted by most as positive or negative. For example, “More than 50% of all fishers lost their job in the last few years” is an objective sentence that clearly describes a negative event. Annotate these sentences as neutral.

Multiple sentiments: In certain instances, opinions about multiple targets are expressed by the author. For instance, the sentence "After some difficulties, the situation got better" encompasses a negative aspect (difficulties at the beginning) but also conveys a positive

outlook. Sentences that represent two or more positions with differing sentiments, for example, "I think this is a good idea, but my friend dislikes it," necessitate a decision on whether one sentiment predominates or if the sentiments are balanced. Sentences of the latter type are to be annotated as neutral.

Rhetorical questions: Rhetorical questions often aim to persuade the reader and should not be interpreted literally. Rather than looking at the superficial meaning, you should try to evaluate the intention behind the rhetorical question instead and make your annotation accordingly. For example, “How should I know?” can be interpreted as negative because the question implies frustration and that the speaker does not feel responsible for the topic the question is referring to.

Different sentiments of author and subject: A different sentiment than the subject being written about can be expressed by authors. For instance, displeasure by the author regarding someone's joy is conveyed in the sentence "Their joy was misplaced." Sentences that indicate some form of disagreement, as in the example, are to be annotated as negative.

1.3.2. Fishery Stance Guideline

The sentences should be carefully read and annotated according to the perspective they articulate regarding the fishery. Each sentence must be classified into one of three categories: Focused on Socio-Economic Viability, Focused on Ecological Health, or Neutral. The classification is determined by whether the sentence emphasizes the needs of the fishery sector or the needs of the marine ecosystem. For simplicity, it is assumed that each sentence conveys a single, primary perspective.

Initially, annotators should determine whether a sentence expresses a clear normative perspective. A sentence is classified as having a perspective if it presents an argument or value judgment regarding the fishery's socio-economic situation or its ecological impact.

For example, the sentence, “New fishing quotas have been established for the upcoming season,” addresses an important fishery topic but does not express a normative judgment. These sentences are to be classified as Neutral and are to be marked with a “1” (excluding the quotation marks). In contrast, the sentence, “The new quotas will make it even harder for small-scale fishers to survive,” clearly prioritizes the economic challenges faced by fishers. This sentence would be classified as Focused on Socio-Economic Viability and is to be marked with a “2” (excluding the quotation marks). Conversely, a sentence like, "The new quotas are insufficient to protect the dwindling cod stock," clearly prioritizes the well-being of the fish population over other concerns. This sentence would be classified as Focused on Ecological Health and is to be marked with a “0” (excluding the quotation marks).

Examples of sentences that express a position on fishery from the corpus are presented in Table S3.

Table S3: Fishery stance sentences. The table presents examples sentences that express a position on fishery in the originall (German language, left column), in translation (English language, middle column) and the assigned fishery stance (right column).

Sentence	Translation	Fishery Stance
Angesichts "dramatisch sinkender" Heringsbestände in der Ostsee hat Bundesagrarministerin Ilse Aigner (CSU) Hilfen für Fischer angekündigt, um die kommenden zwei Jahre zu überbrücken.	In view of “dramatically declining” herring stocks in the Baltic Sea, Federal Minister of Agriculture Ilse Aigner (CSU) has announced aid for fishermen to bridge the next two years.	Socio-Economic Viability
Greenpeace machte die Trawler der „Industrieflotten“ hauptsächlich für die Überfischung in der Ostsee verantwortlich.	Greenpeace held the trawlers of the “industrial fleets” primarily responsible for overfishing in the Baltic Sea.	Ecological Health
Auch Hamburgs Bürgermeister Olaf Scholz (SPD) begrüßte die Reform und wies auf die historische Bedeutung Hamburgs als Standort für die Fischerei hin.	Hamburg’s Mayor Olaf Scholz (SPD) also welcomed the reform and pointed out the historical significance of Hamburg as a hub for the fishing industry.	Socio-Economic Viability
Greenpeace kritisierte, dass sich die EU-Minister "erneut dem Druck der Fischereilobby" gebeugt hätten.	Greenpeace criticized that the EU ministers had once again succumbed to the pressure of the fishing lobby.	Ecological Health

Der Vorsitzende des Landesfischereiverbandes, Lorenz Marckwardt, begrüßte die Aktion.	The chairman of the state fisheries association, Lorenz Marckwardt, welcomed the action.	Socio-Economic Viability

Special Cases

Mixed Stances: If a sentence contains elements of both a socio-economic and an ecological perspective, determine which perspective clearly predominates. If a clear majority is present, annotate the sentence according to the dominant perspective (i.e., either 'Focused on Socio-Economic Viability' or 'Focused on Ecological Health'). However, if the sentence presents a truly balanced view or an unresolved dilemma, it should be annotated as Neutral.

Positions from Fishers themselves: When fishers or their representatives speak, their statements should be classified as Focused on Socio-Economic Viability, even if the sentiment expressed is one of concern or dissatisfaction. This is because such statements inherently articulate the challenges and needs of the fishery sector. For example, the sentence, “The chairman of the state fisheries association expressed his concern about the current situation,” should be annotated as Focused on Socio-Economic Viability, as it represents the perspective and challenges of the fishery.

1.3.3. Topic Annotation Guideline

Each sentence should be carefully read and annotated according to its primary topic. Each sentence must be classified into one of five topics: (i) Regulatory fishery law, (ii) Scientific (governance) advice, (iii) Fisheries subsidies and institutional support, (iv) Environmental and nature conservation, or (v) Others. The classification is determined by the main subject matter addressed in the sentence.

Definition of Categories

(i) Regulatory fishery law: Sentences that discuss the regulatory fishery law, including quota allocation, fishing restrictions, and other regulatory policy measures directly related to fisheries governance. These sentences are to be marked with a “1” (excluding the quotation marks).

(ii) Scientific (governance) advice: Sentences, in which references to scientific advice for fishery governance, such as stock assessments or other forms of fishery related policy recommendations, can be found. These sentences are to be marked with a “2” (excluding the quotation marks).

(iii) Fisheries subsidies and institutional support: Sentences that refer to financial, technical, or institutional assistance provided to fishers, fishery cooperatives, or related industries. Additionally, this category includes references to commercial fisheries and market-related aspects of the fishery sector. These sentences are to be marked with a “3” (excluding the quotation marks).

(iv) Environmental and nature conservation: Sentences that address ecological concerns related to fisheries, marine conservation efforts, or environmental sustainability. This includes topics such as overfishing, habitat destruction, biodiversity conservation, and climate change impacts on fisheries. These sentences are to be marked with a “4” (excluding the quotation marks).

(v) Others: Sentences that do not clearly fit into any of the above categories, including general news reports, human interest stories, historical accounts, or statements without a strong thematic focus on stocks, aid, or sustainability. These sentences are to be marked with a “5” (excluding the quotation marks).

Table S4: Topic sentences. The table presents examples of topic sentences in the original (German language, left column), in translation (English language, middle column) and the chosen topic (right column).

Sentence	Translation	Topic
Der Internationale Rat für Meeresforschung (ICES) habe empfohlen, die Fangquoten für das Jahr 2011 in der westlichen Ostsee um sechs Prozent zu erhöhen, teilte der Verband der Deutschen Kutter- und Küstenfischer gestern in Hamburg mit.	The International Council for the Exploration of the Sea (ICES) has recommended increasing fishing quotas for the western Baltic Sea by six percent for the year 2011, the Association of German Cutter and Coastal Fishermen announced yesterday in Hamburg.	Scientific (governance) advice
Die Fischer und das EuroBaltic-Fischwerk in Sassnitz erwarten mit dem Siegel des Marine Stewardship Council eine bessere Vermarktbarkeit des Herings und höhere Erlöse.	The fishers and the EuroBaltic fish factory in Sassnitz have been awarded the Marine Stewardship Council's seal, improving the marketability of herring and leading to higher revenues.	Fisheries subsidies and institutional support
Nach Ansicht der Umwelt-NGOs steht der Meeresschutz in Nord- und Ostsee bisher nur auf dem Papier.	According to environmental NGOs, marine protection in the North Sea and Baltic Sea exists only on paper so far.	Environmental and nature conservation
Bundesagrarministerin Ilse Aigner (CSU) ist auf Sommerreise im Nordosten der Republik.	Federal Minister of Agriculture Ilse Aigner (CSU) is on a summer tour in the northeast of the country.	Others
Die europäischen Fischereiminister einigten sich am gestrigen Dienstag in Luxemburg auf die Ostsee-Fangquoten für kommendes Jahr.	The European fisheries ministers agreed on next year's Baltic Sea fishing quotas yesterday, Tuesday, in Luxembourg.	Regulatory fishery law
Die Stimmung in der größten Fischereigenossenschaft des Landes liege am Boden, so Schütt.	The mood in the largest fisheries cooperative in the country is at rock bottom, said Schütt.	Fisheries subsidies and institutional support
Vor allem durch Fischerei mit Grundschleppnetzen „erreichen die Lebensräume des Meeresbodens und die Meeresfische insgesamt nicht den guten Umweltzustand", bestätigte das Bundesumweltministerium auf Anfrage der Grünen-Abgeordneten Steffi Lemke.	Fishing with bottom trawls in particular means that "the habitats of the seabed and marine fish as a whole do not achieve good environmental status," confirmed the Federal Ministry for the Environment in response to a request from Green Party MP Steffi Lemke.	Environmental and nature conservation

Sentences with Multiple Topics: If a sentence addresses multiple topics, the annotator should choose the most prominent one. If no single topic is dominant, the sentence should be classified under "Others."

1.4. Model Evaluations

1.4.1. Sentiment Classification

Figure S1 displays the confusion matrix for the sentiment test dataset. Each row of the confusion matrix represents the instances in an actual class, while each column represents the instances in a predicted class. The main diagonal contains all instances which were correctly classified. Notably, despite the scarcity of positive sentences in the training dataset, the model effectively learns to classify them: out of 39 positive sentences in the test set, 26 (66,67%) are accurately labeled as positive. The performances for the neutral and negative sentences are higher due to the larger number of these sentences available for training the model. Overall, the model produces an accuracy of 89% on the test dataset, showing that it is well-suited for the sentiment classification task in our dataset.

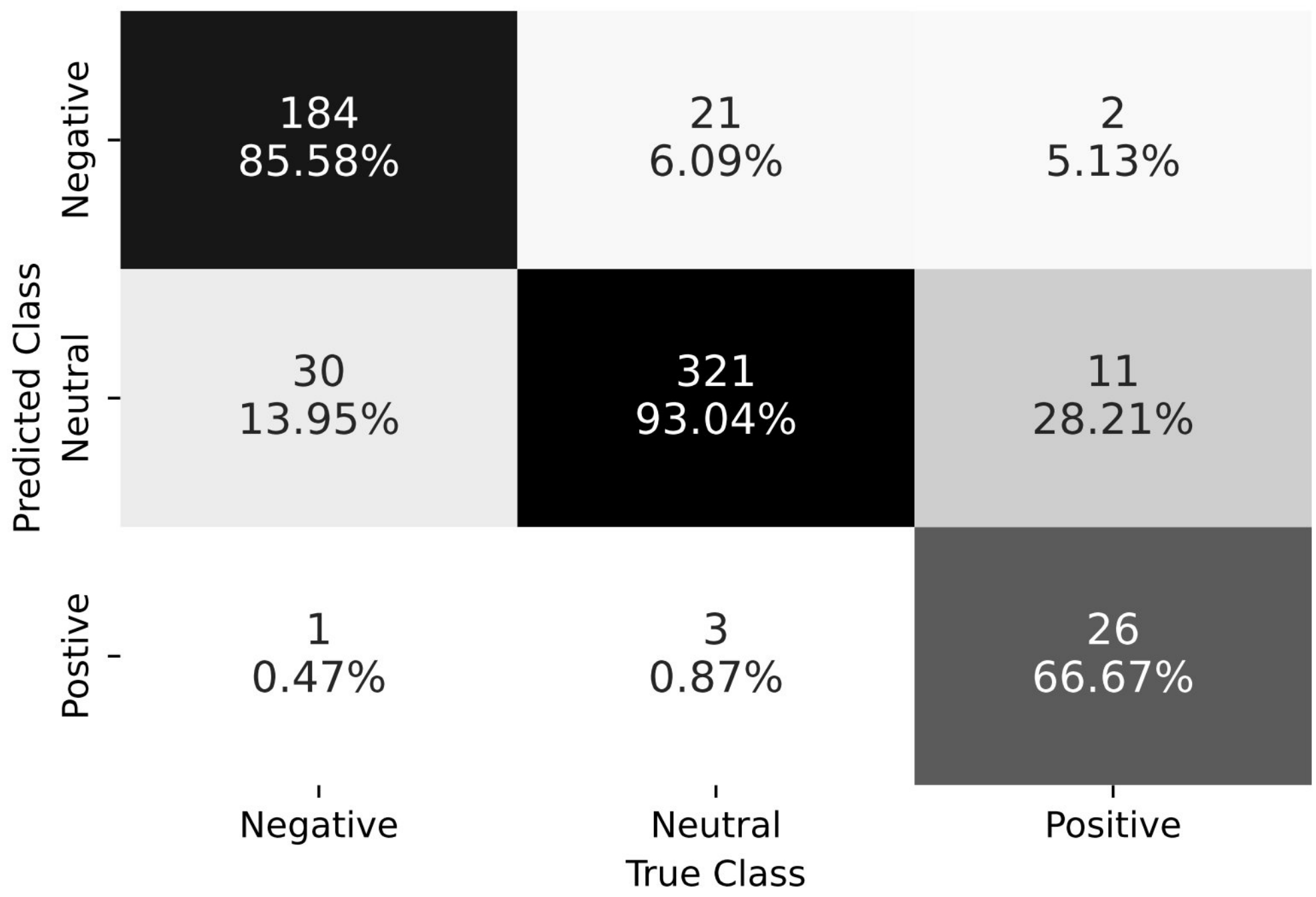

Figure S1: Confusion matrix for the sentiment test set. Each row of the confusion matrix represents the instances in an actual class, while each column represents the instances in a predicted class. The main diagonal contains all instances which were correctly classified. The percentages in each cell represent the proportion of predictions relative to the actual class, highlighting the model's performance in distinguishing between negative, neutral, and positive sentiments.

1.4.2. Fishery Stance Classification

Figure S2 displays the confusion matrix for the fishery stance test dataset. The model performs well across all three stance categories. 50 out of 69 sentences focused on ecological health (72.46%) are correctly classified, while neutral sentences and sentences focused on ecological health achieve accuracy rates of 93.78% and 89.38%, respectively.

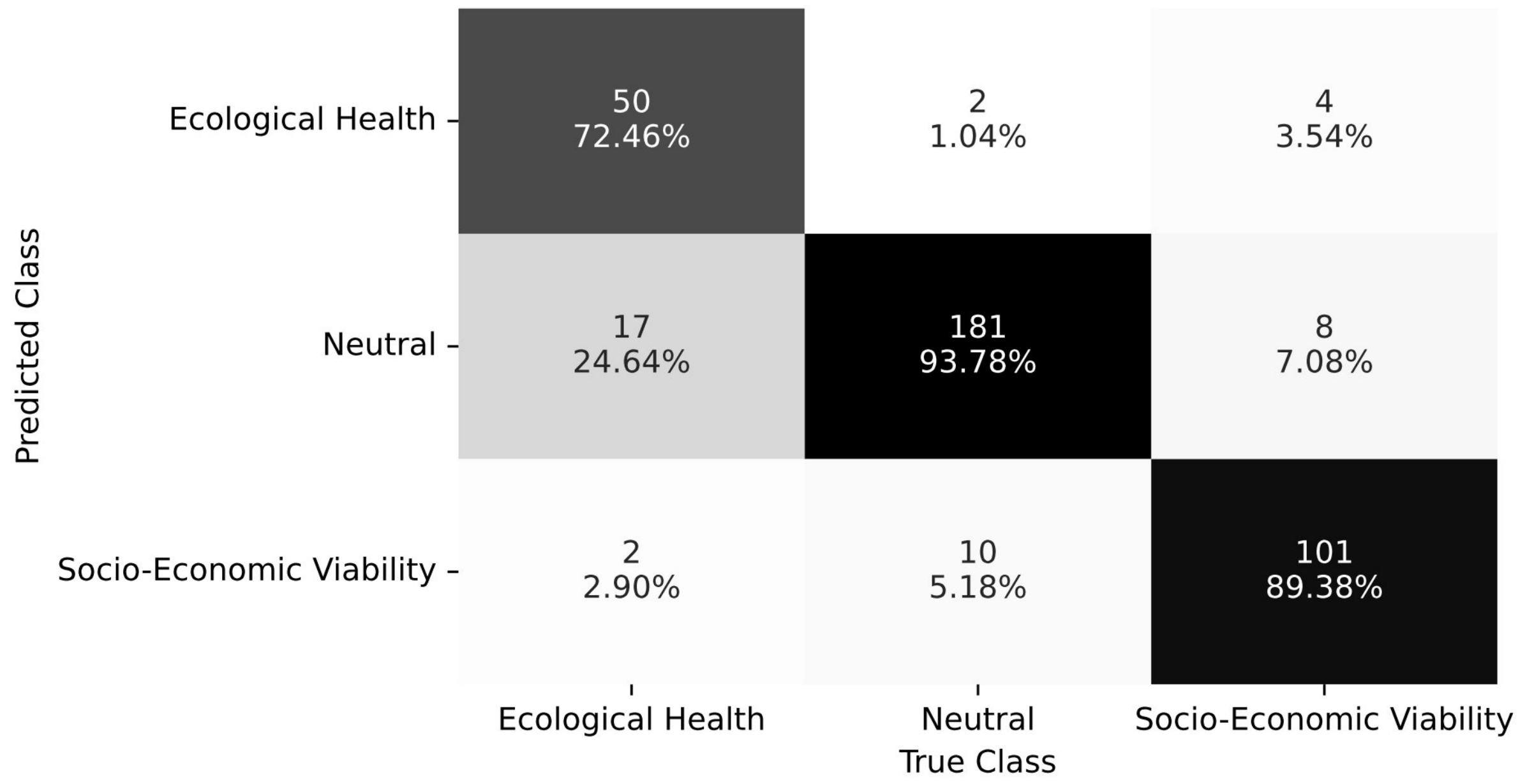

Figure S2: Confusion matrix for the fishery stance test set. Each row of the confusion matrix represents the instances in an actual class, while each column represents the instances in a predicted class. The main diagonal contains all instances which were correctly classified. The percentages in each cell represent the proportion of predictions relative to the actual class, highlighting the model's performance in distinguishing between the three stance categories: Focused on Socio-Economic Viability, Neutral, and Focused on Ecological Health.

1.4.3. Topic Classification

Figure S3 displays the confusion matrix for the topic classification test dataset. Each row of the confusion matrix represents the instances in an actual class, while each column represents the instances in a predicted class. The main diagonal contains all instances that were correctly classified. The model demonstrates strong performance in distinguishing between topics, particularly for “Fisheries subsidies and institutional support” where 101 out of 118 sentences (85.59%) were correctly classified. “Environmental and nature conservation” also shows a high accuracy of 79.84%, with 103 out of 129 sentences classified correctly.

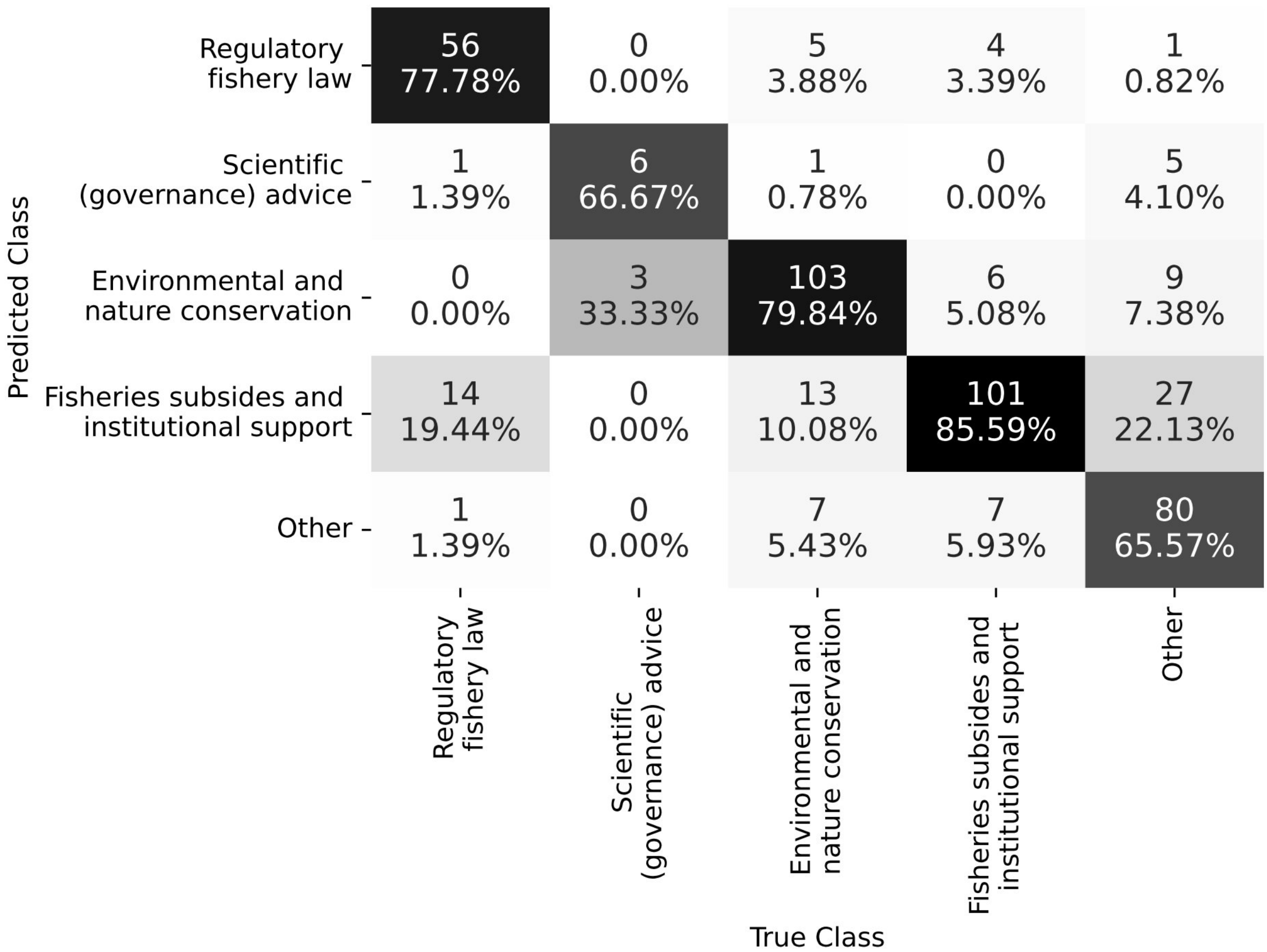

Figure S3: Confusion matrix for the topic test set. Each row of the confusion matrix represents the instances in an actual class, while each column represents the instances in a predicted class. The main diagonal contains all instances which were correctly classified. The percentages in each cell represent the proportion of predictions relative to the actual class, highlighting the model's performance in distinguishing between the four topics.

2. Supporting Summary Statistics

2.1. Article Publications Over Time

Figure S4 illustrates the number of published articles each year from 2009 to 2021. It shows a gradual upward trend over the years, with a notable increase in the volume of articles starting from around 2014, peaking in 2021. This phenomenon can be attributed primarily to the gradual transition of newspapers towards digital archives that are accessible online. It therefore does not indicate an increasing relevance of the German Baltic fishery in the public discourse.

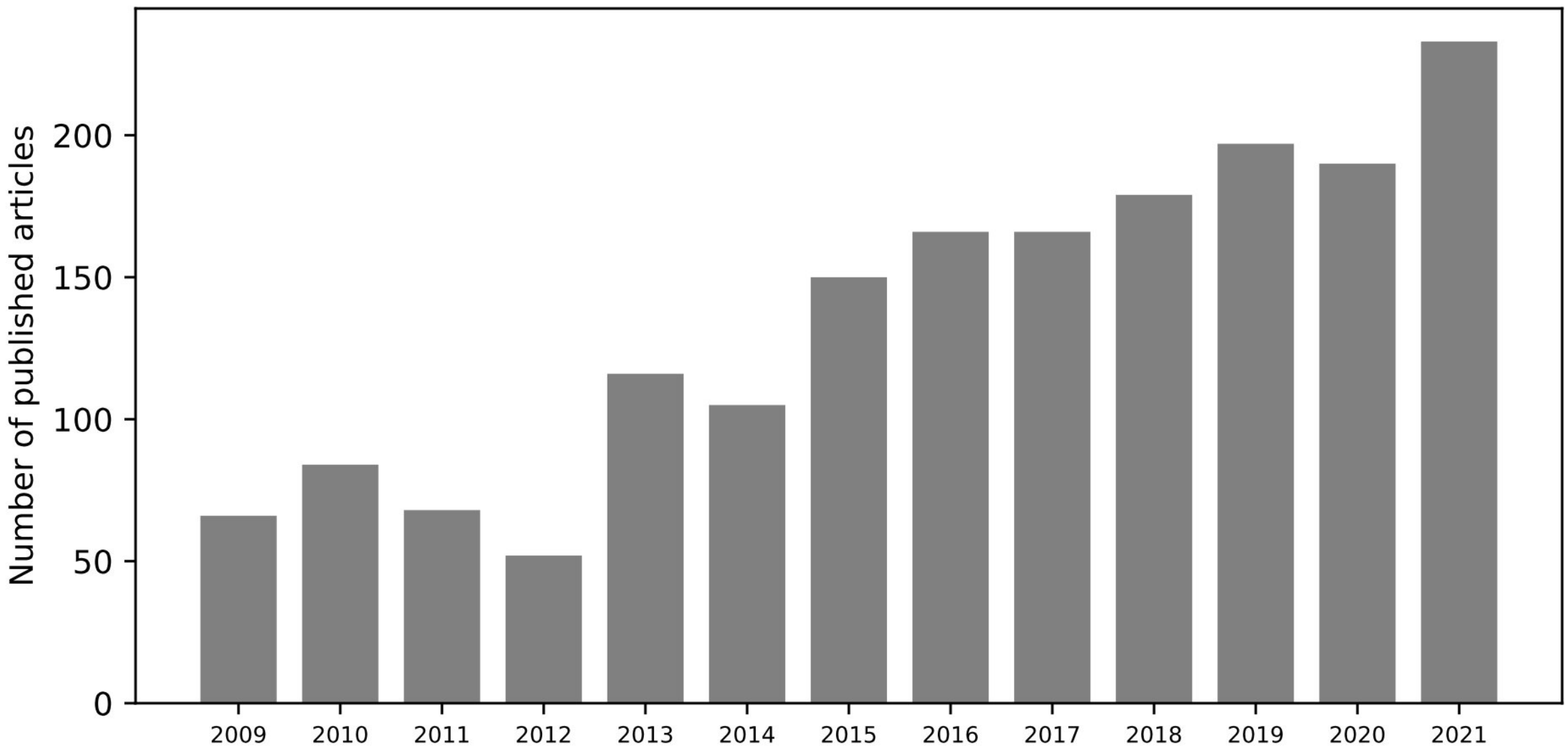

Figure S4. Number of published articles related to the German Baltic fishery. The grey bars show the number of articles in our dataset based on their publication within a calendar year.

2.2. Stakeholder Selection Threshold

The tables below provide a detailed description of our data set. Table S5 includes all newspapers in our sample that reported at least three times about the German Baltic Fisheries. The newspapers with the most articles are regional, coming from the federal states of Schleswig-Holstein and Mecklenburg-Vorpommern which are on the Baltic Seas coast (e.g., Ostsee Zeitung, Schleswig-Holstein Zeitung, Hamburger Abendblatt, Schweriner Volkszeitung).

Table S5: List of newspaper that reported at least three times about the German Baltic Fisheries. N is the number of times in our sample a newspaper article reported about the German Baltic Sea fisheries.

Newspaper	N	Newspaper	N
OstseeZeitung	505	WWF	10
SHZ	244	Lebensmittel Zeitung	10
Hamburger Abendblatt	183	Nordwest-Zeitung	9
SVZ	171	ots	8
Die Welt	96	Täglicher Hafenbericht	7
dpa RegioLine	58	Aachener Nachrichten	7
taz	52	Deutschlandfunk	6
Süddeutsche Zeitung	45	NTV	6
dpa-AFX	40	ProPlanta	6

LN	29	Fehmarn24	5
AFP	21	ARD	5
Frankfurter Rundschau	18	General-Anzeiger	5
Stuttgarter Nachrichten	17	Rheinische Post	4
Mitteldeutsche Zeitung	17	Berliner Kurier	4
Berliner Zeitung	15	Tagesschau	4
dpa Infoline Politik und Wirtschaft etc.	15	KN	4
LZ.net	15	Focus	4
Stuttgarter Zeitung	14	Merkur	3
ZEIT	14	Aachener Zeitung	3
Nordkurier	13	Greenpeace	3
SPIEGEL	13	Kölnische Rundschau	3
Die Welt kompakt	13	SWR	3
Der Tagesspiegel	12	Agrar Presseportal	3
Die Welt Hamburg	12	Mynewsdesk	3
SPIEGEL ONLINE	11		

Table S6a reports the stakeholder organizations mentioned at least 5 times in our sample. The two organizations most frequently mentioned are the WWF (N=656; stakeholder group: eNGO) and the European Commission (N=646; stakeholder group: “Politics & Public Authorities”). This is followed by Greenpeace (N=516; stakeholder group: “eNGO”), the International Council for the Exploration of the Sea (N=445; stakeholder group: “Science”) and Thünen Institute for Baltic Sea Fisheries (N=321).

Table S6b reports the individual stakeholders mentioned at least 5 time in our sample. The most often mentioned stakeholder is Christopher Zimmermann (N=663; stakeholder group: “Science”; Thuenen Institute for Baltic Sea Fisheries). He is followed by Michael Schütt (N= 373; stakeholder group: “Fisheries”; fisheries cooperative Peenemündung Freest) and Till Backhaus (N=296; stakeholder group: “Politics & Public Authorities”, member of the German party SPD).

Overall, we identified 60 organizations (5884 total occurrences) and 183 individuals (6567 total occurrences) which occur at least 5 time in our dataset of which 37 different organizations (5623 total occurrences) and 79 individuals (5534 total occurrences) occur at least 20 times in our dataset.

Table S6c lists the stakeholder groups by governance level with at least 20 occurrences. We used 20 as the threshold for our analysis to examine stakeholders who demonstrate a consistent and persistent presence in the news over time. Additionally, we found that lowering the threshold to 5 occurrences does not meaningfully impact our results. Results for the threshold of 5 occurrences are available upon request.

Table S6a: List of organizations with at least 5 occurrences. The organizations are categorized by their stakeholder group ("eNGO", "Fisheries, Politics & Public Authorities", and "Science") and governance levels (GL, subnational, national and international). The "Mentions" column indicates the number of times each organization was mentioned in the sample.

GL	Name	Mentions
**eNGO**		
National	WWF	656
	Greenpeace	516
	Marine Stewardship Council	284
	Naturschutzbund (*Nature and Biodiversity Conservation Union*)	125
	Deutsche Umwelthilfe (*Environmental Action Germany*)	72
	Deutscher Naturschutzring (*German League for Nature and Environment*)	17
	Peta	14
	BUND	6
	Animal Rights Watch	5
International	Oceana	78
	Initiative Our Fish	27
	Aquaculture Stewardship Council	11
	Deepwave	7
**Fisheries**		
Subnational	Fischereigenossenschaften (F*ishing cooperatives*)	280
	Landesverband der Kutter- und Küstenfischer Mecklenburg-Vorpommern (*State Association of Cutter and Coastal Fishermen Mecklenburg-Vorpommern*)	221
	Landesfischereiverband Schleswig-Holstein (*State Fishing Association Schleswig-Holstein*)	147
	Landesfischereiverband Mecklenburg-Vorpommern (*State Fishing Association Mecklenburg-Vorpommern*)	51
	Euro-Baltic	57
	Küstenfischer Nord (*Coastal Fisherman North*)	17
	Holmer Fischerzunft (*Holm fishing guild*)	10
	Fischereiverein Flensburg (*Flensburg Fishing Club*)	6
National	Deutscher Fischerei-Verband *(German Fishing Association*)	86
	Fisch-Informationszentrum Hamburg (*Fish Information Center*	20

	Hamburg)	
	Deutscher Hochseefischerei-Verband (*German Deep Sea Fishing Association*)	15

Politics & Public Authorities

Subnational	Landesamt für Landwirtschaft, Lebensmittelsicherheit und Fischerei Schleswig-Holstein (*Schleswig-Holstein State Office for Agriculture, Food Safety and Fisheries*)	51
	Landesamt für Umwelt , Naturschutz und Geologie Schleswig-Holstein (*Schleswig-Holste State Office for the Environment, Nature Conservation and Geology)*	11
National	Bündnis 90 / Die Grünen (*German Party*)	160
	Bundeamt für Naturschutz (*Federal Agency for Nature Conservation*)	144
	CSU (*German Party)*	120
	Bundesanstalt für Landwirtschaft und Ernährung (*Federal Agency for Agriculture and Food*)	67
	Bundesamt für Seeschifffahrt und Hydrographie (*Federal Maritime and Hydrographic Agency of Germany)*	45
	Bundesministerium für Umwelt, Naturschutz, nukleare Sicherheit und Verbraucherschutz (*Federal Ministry for the Environment, Nature Conservation, Nuclear Safety and Consumer Protection)*	44
	Umweltbundesamt (*Federal Environment Agency*)	22
	FDP (*German Party)*	22
	AFD (*German Party)*	16
	SSW (*German Party)*	6
International	European Commission	646
	European Parliament	121
	Helsinki Commission	36
	European Court of Justice	23
	European Environment Agency	14

Science

National	Thünen-Institut für Ostseefischerei (*Thünen Institute for Baltic Sea Fisheries*)	321
	Deutsches Meeresmuseum (*German Maritime Museum)*	47
	Ostsee Info-Center	40
	Geomar Helmholtz-Zentrum für Ozeanforschung (*Geomar Helmholtz Center for Ocean Research)*	35
	Leibniz-Institut für Ostseeforschung (*Leibniz Institute for Baltic Sea Research*)	19
	Centrum für Erdsystemforschung und Nachhaltigkeit (*Center for Earth System Research and Sustainability*)	15
	Deutschen Zentrums für integrative Biodiversitätsforschung Halle-Jena-Leipzig (*German Center for Integrative Biodiversity Research Halle-Jena-Leipzig*)	11
	Gesellschaft zum Schutz der Meeressäugetiere (*Society for the Protection of Marine Mammals)*	6
International	International Council for the Exploration of the Sea	445
	Scientific Reports	16

Table S6 b: List of individual stakeholders with at least 5 occurrences. The stakeholders are categorized by their stakeholder groups ("eNGO", "Fisheries", "Politics & Public Authorities", and "Science") and governance levels (GL, subnational, national, and international). The "Institution" column indicates with which organization each stakeholder is affiliated, while the "Mentions" column indicates the number of times each individual was mentioned in the sample.

GL	Name	Institution	Mentions
**eNGO**			
Subnational	Jochen Lamp	WWF	37
National	Thilo Maack	Greenpeace	198
	Stella Nemecky	WWF	146
	Gabriele Dederer	WWF	22
	Onno Groß	Deepwave	22
	Kim Detloff	NABU	21
	Heike Vesper	WWF	20
	Sandra Schöttner	Greenpeace	13
	Nadja Ziebarth	BUND	11
	Ingo Ludwichowski	NABU	10
	Karoline Schacht	WWF	8
	Sascha Müller-Kraenner	Deutsche Umwelthilfe (*Environmental Action Germany*)	8
	Britta König	WWF	7
	Olaf Tschimpke	NABU	7
	Hermann Schultz	NABU	6
International	Philipp Kanstinger	WWF	21
	Pascale Moehrle	Oceana	14
	Lasse Gustavsson	Oceana	6
**Fisheries**			
Subnational	Michael Schütt	General manager of fisheries cooperative „Peenemündung-Freest e.G.", vice chair of Fishing Association Mecklenburg-Vorpommern	373
	Lorenz Marckwardt	Chair of State Fishing Association Schleswig-Holstein	175
	Uwe Richter	General manager of „Doggerbank Seefischerei GmbH"	166
	Norbert Kahlfuss	Chair of State Association of Cutter and Coastal Fishermen Mecklenburg-Vorpommern	156
	Ralph Krehl	Own business	115
	Benjamin Schmöde	General manager of fisheries cooperative Fehmarn, vice chair of State Fishing Association Schleswig-Holstein	104
	Jörn Ross	Own business	84
	Dirk Baumann	Own business	83
	Horst-Dieter Hansen	Own business	59
	Ilona Schreiber	General manager fisheries cooperative Wismarbucht e. G.; chair of State	52

	Association of Cutter and Coastal Fishermen Mecklenburg-Vorpommern	
Gerhard Draasch	Own business; Executive Board of „Fördervereins „Fischereigeschichte Möltenort e.V.“	52
Ingo Pinnow	Own business	51
Dirk Sander	Chair of State Association of Cutter and Coastal Fishermen Mecklenburg-Vorpommern	51
Uwe Krüger	Own business	44
Günter Grothe	Chair of State Association of Cutter and Coastal Fishermen Mecklenburg-Vorpommern	42
Dieter Hullmann	Own business	39
Uwe Dunkelmann	Own business	39
Kai Dunkelmann	Own business	38
Henry Diedrich	Own business	37
Jürgen Krieger	Own business	36
Matthias Nanz	Own business	36
Martin Saager	Own business	36
Silvio Mundt	Own business	36
Jörg Nadler	Own business	35
André Grählert	Own business	32
Uwe Pinkis	Own business	31
Klaus Pinkis	Own business	27
Gretel Flindt	Chair of State Fishing Association Schleswig-Holstein	27
Martin Lange	Own business	24
Erwin Mundt	Own business	24
Horst Ross	Own business	23
Christian Ross	Own business	22
Thomas Koldevitz	Own business	21
Ulrich Elsner	General manager fisheries cooperative „Küstenfischer Nord eG Heiligenhafen“	21
Wolfgang Albrecht	Chair of Fisheries Protection Association Schleswig-Holstein	21
Andreas Zirkler	Own business	21
Horst Grählert	Own business	21
Gunnar Gerth-Hansen	Own business	18
Heiko Gores	Own business	17
Carsten Foth	Own business	17
Horst Sorge	Own business	17
Elvira Rothe	Wismar fishing cooperative	17
Stefan Kübart	Own business	17
Björn Michalak	Own business	15
Steffen Schnorrenberg	Own business	15
Hartmut Hübert	Own business	15
Jörg Bernier	Own business	14
Ulrich Paetsch	Own business	14
Frank Steinbusch	Own business	14
Rüdiger Krüger	Own business	14
Martin Heiden	Own business	13
Hans-Joachim Bull	Own business	13
Peter Jöhnk	Own business	13

	Peter Wolff	Own business	13
	Paul Keppler	Own business	12
	Andreas Berthold	Operations manager at „Euro-Baltic“	12
	Mike Hilger	Own business	12
	Marc Richter	Own business	11
	Roberto Keppler	Own business	11
	Detlef Diedrich	Own business	10
	Ulrich Karnatz	Own business	10
	Jens-Peter Mahrt	Own business	10
	Elke Horndasch-Petersen	President of State Fishing Association Schleswig-Holstein	9
	Jens Kraeft	Own business	9
	Thomas Jandt	Own business	9
	Paul Hübner	Own business	9
	Kay Briesewitz	Own business	8
	Günter Klever	Own business	8
	Paul Zipolt	Own business	8
	Jan Wienholz	Own business	7
	Eike Düwel	Own business	6
	Jörg Perschke	Own business	6
	Maik Seemann	Own business	6
	Roland Scheller	Own business	6
	Torsten Labahn	Own business	6
	Sven Detlefsen	Own business	5
	Dennis Freitag	Own business	5
	Lasse Mahrt	Own business	5
	Max Riegert	Own business	5
	Björn Mahrt	Own business	5
	Erik Meyer	Own business	5
	David Graf	Own business	5
	Armin Laß	Own business	5
	Günther Sakuth	Secretary at Flensburg Fishing Association	5
	Wolfgang Schmitz	Own business	5
National	Peter Breckling	President of German Fishing Association	88
	Claus Ubl	Media office at German Fishing Association	65
	Holger Ortel	President of German Fishing Association	35
	Matthias Keller	General manager of Fish Information Center Hamburg	35
	Kai-Arne Schmidt	General manager of „Kutterfisch-Zentrale GmbH“	33
**Politics & Public Authorities**			
Subnational	Holger Klink	Mayor of the town „Strande“	9
	Petra Nicolaisen	CDU (*German Party)*	6
	Clemens Teschendorf	Administration spokesperson of the city „Flensburg“	5
	Erich Skrey	Landesamt für Landwirtschaft, Ländliche Räume & Umwelt Schleswig-Holstein (*State Office for Agriculture, Rural Areas & Environment Schleswig-Holstein*)	5
	Wolfgang Vogel	Director of „Landesamtes für Natur und Umwelt Schleswig-Holstein“	5
	Antje Hückstädt	Municipal representative of the town	5

		„Born“	
National	Till Backhaus	SPD (*German Party*)	296
	Robert Habeck	Bündnis 90 / Die Grünen (*German Party*)	191
	Julia Klöckner	CDU (*German Party)*	183
	Christian Schmidt	SPD (*German Party*)	166
	Ilse Aigner	CSU (*German Party)*	103
	Henning von Nordheim	Bundesministerium für Umwelt, Naturschutz, nukleare Sicherheit und Verbraucherschutz (*Federal Ministry for the Environment, Nature Conservation, Nuclear Safety and Consumer Protection)*	76
	Jan Philipp Albrecht	Bündnis 90 / Die Grünen (*German Party*)	54
	Beate Jessel	Bundesministerium für Umwelt, Naturschutz, nukleare Sicherheit und Verbraucherschutz (*Federal Ministry for the Environment, Nature Conservation, Nuclear Safety and Consumer Protection)*	28
	Steffi Lemke	Bündnis 90 / Die Grünen (*German Party*)	27
	Angela Merkel	CDU (*German Party)*	21
	Daniel Günther	CDU (*German Party)*	21
	Robert Kloos	Bundesanstalt für Landwirtschaft und Ernährung (*Federal Agency for Agriculture and Food*)	18
	Barbara Hendricks	SPD (*German Party*)	15
	Juliane Rumpf	CDU (*German Party)*	14
	Svenja Schulze	Bündnis 90 / Die Grünen (*German Party*)	13
	Friedrich Ostendorff	Bündnis 90 / Die Grünen (*German Party*)	11
	Jürgen Buchwald	Umweltministerium Mecklenburg-Vorpommern (*Ministry of the Environment of Mecklenburg-Vorpommern*)	11
	Peter Harry Carstensen	CDU (*German Party)*	10
	Jochen Flasbarth	SPD (*German Party*)	9
	Ingo Gädechens	CDU (*German Party)*	8
	Johannes Callsen	CDU (*German Party)*	8
	Claus Tantzen	Ministerium für Landwirtschaft und Umwelt Mecklenburg-Vorpommern (*Ministry of Agriculture and Environment of Mecklenburg-Vorpommern*)	8
	Ralf Borschke	AFD (*German Party)*	8
	Rasmus Andresen	Bündnis 90 / Die Grünen (*German Party*)	7
	Silke Schneider	Ministerium für Landwirtschaft und Umwelt Mecklenburg-Vorpommern (*Ministry of Agriculture and Environment of Mecklenburg-Vorpommern*)	7
	Simone Oldenburg	Die Linke (*German Party*)	6
	Hagen Reinhold	FDP (*German Party*)	6
	Bettina Hagedorn	SPD (*German Party*)	6
	Sönke Rix	SPD (*German Party*)	5
	Philipp Amthor	CDU (*German Party)*	5
	Karsten Kolbe	Die Linke (*German Party*)	5
International	Maria Damanaki	European Commission	98
	Werner Kuhn	EU Parliament (CDU)	91
	Karmenu Vella	European Commission	91

	Ulrike Rodust	European Parliament (SPD)	87
	Niclas Herbst	European Parliament (FDP)	58
	Claus Müller	Hanseoffice	23
	Virginijus Sinkevicius	European Commission	14
	Joe Borg	European Commission	13
	Siim Kiisler	Estland Government	6
**Science**			
National	Christopher Zimmermann	Director of „Thünen-Institut für Ostseefischerei“ (*Thünen Institute for Baltic Sea Fisheries*)	663
	Rainer Froese	Scientist at „Geomar Helmholtz-Zentrum für Ozeanforschung“ (*Geomar Helmholtz Center for Ocean Research)*	65
	Michael Dähne	Scientist at „Deutsches Meeresmuseum“ (*German Maritime Museum)*	41
	Christian Möllmann	Scientist at „University Hamburg“	38
	Cornelius Hammer	Scientist at „Thünen-Institut für Ostseefischerei“ (*Thünen Institute for Baltic Sea Fisheries*)	34
	Reinhold Hanel	Scientist at „Thünen-Institut für Ostseefischerei“ (*Thünen Institute for Baltic Sea Fisheries*)	29
	Daniel Stepputtis	Scientist at „Thünen-Institut für Ostseefischerei“ (*Thünen Institute for Baltic Sea Fisheries*)	25
	Patrick Polte	Scientist at „Thünen-Institut für Ostseefischerei“ (*Thünen Institute for Baltic Sea Fisheries*)	19
	Florian Peine	Scientist at „Landesforschungsanstalt Mecklemburg-Vorpommern“ *(Mecklemburg-Western Pomerania State Research Institute)*	18
	Harald Benke	Scientist at „Deutsches Meeresmuseum“ (*German Maritime Museum)*	14
	Harry Strehlow	Scientist at „Thünen-Institut für Ostseefischerei“ (*Thünen Institute for Baltic Sea Fisheries*)	11
	Thorsten Reusch	Scientist at „Geomar Helmholtz-Zentrum für Ozeanforschung“ (*Geomar Helmholtz Center for Ocean Research)*	11
	Christoph Stransky	Scientist at „Thünen-Institut für Fischereiökologie“ (*Thünen Institute for Baltic Sea Fisheries*)	11
	Andreas Pfander	Scientist (*Ostsee Info-Center*)	10
	Christian von Dorrien	Scientist at „Thünen-Institut für Ostseefischerei“ (*Thünen Institute for Baltic Sea Fisheries*)	10
	Anja Gallus	Scientist at „Deutsches Meeresmuseum“ (*German Maritime Museum)*	10

	Gerd Kraus	Scientist at „Thünen-Institut für Ostseefischerei“ (*Thünen Institute for Baltic Sea Fisheries*)	9
	Carsten Kühn	Scientist at „Landesforschungsanstalt Mecklemburg-Vorpommern“ *(Mecklemburg-Western Pomerania State Research Institute)*	8
	Robert Arlinghaus	Scientist at „Humbolt-Universität Berlin“	8
	Uwe Krumme	Scientist at „Thünen-Institut für Ostseefischerei“ (*Thünen Institute for Baltic Sea Fisheries*)	7
	Jamileh Javidpour	Scientist at „Geomar Helmholtz-Zentrum für Ozeanforschung“ (*Geomar Helmholtz Center for Ocean Research)*	6
	Catriona Clemmesen	Scientist at „Geomar Helmholtz-Zentrum für Ozeanforschung“ (*Geomar Helmholtz Center for Ocean Research)*	5
	Markus Meier	Scientist at „Leibniz-Institut für Ostseeforschung“ (*Leibniz Institute for Baltic Sea Research*)	5
International	Thorsten Blenckner	Scientist at „Stockholm University“	29
	Boris Worm	Scientist at „University British Columbia“	12

Table S6c: List of stakeholder groups by governance level (GL) with at least 20 occurrences. Stakeholders are categorized by stakeholder group (“eNGO”, “Fisheries”, “Politics & Public Authorities”, and “Science”) and governance levels (GL, subnational, national, and international). The "Individuals" column indicates the number of mentions of individual stakeholders, while the "Organizations" column indicates the number of mentions of stakeholder organizations. The "Total" column represents the combined occurrences of individuals and organizations for each governance level and overall.

GL	Individuals	Organizations	Total
**eNGO**			
Subnational	37	0	37
National	429	1653	2082
International	21	105	126
Total	487	1758	2245
**Fisheries**			
Subnational	2254	756	3010
National	256	106	362
International	0	0	0
Total	2510	862	3372
**Politics & Public Authorities**			
Subnational	0	51	51
National	1165	1217	2382
International	448	826	1274
Total	1613	2094	3707
**Science**			
Subnational	0	0	0
National	895	466	1338
International	29	443	495
Total	924	909	1833
**Overall**			
Subnational	2291	807	3098
National	2745	3419	6164
International	498	1397	1895
Total	5534	5623	11157

2.3. Summary Statistics

Table S7: Newspaper sentiment by fish species. The table presents summary statistics for the newspaper sentiment indices for sentences related to herring, cod, and for the full dataset. The table includes the means of the sentiment indices, standard deviation (S.D.), minimum (Min.), and maximum (Max.). The yearly data is based on the ‘fishery years’. The quarterly values are calculated based on quarters defined by the ‘fishery years’.

**Quarterly**	**Mean**	**S.D.**	**Min.**	**Max.**
Overall	-0.249	0.063	-0.404	-0.108
Herring	-0.240	0.076	-0.444	-0.087
Cod	-0.251	0.067	-0.444	-0.080
**Yearly**				
Overall	-0.249	0.044	-0.302	-0.153
Herring	-0.241	0.054	-0.317	-0.131
Cod	-0.255	0.047	-0.331	-0.163

Table S8: Newspaper sentiment by stakeholder group. The table presents summary statistics for the newspaper sentiment indices for sentences that include a stakeholder belonging to one of the groups. The table includes the mean sentiment scores, standard deviation (S.D.), minimum (Min.), and maximum (Max.) values for individuals, organizations, and combined individuals and organizations. The stakeholder groups include “Politics & Public Authorities”, “Fisheries”, “Science”, and “eNGO”, as well as a combined "All Groups" category. The sentiment indices are based on the ‘fishery years’ and are calculated on a yearly basis.

**Individuals**	**Mean**	**S.D.**	**Min.**	**Max.**
Politics & Public Authorities	-0.282	0.087	-0.425	-0.145
Fisheries	-0.307	0.068	-0.419	-0.163
Science	-0.366	0.102	-0.446	-0.086
eNGO	-0.470	0.197	-1.000	-0.231
All Groups	-0.320	0.050	-0.396	-0.233
**Organizations**				
Politics & Public Authorities	-0.245	0.084	-0.362	-0.093
Fisheries	-0.341	0.135	-0.500	-0.045
Science	-0.236	0.122	-0.404	-0.045
eNGO	-0.371	0.132	-0.542	-0.141
All Groups	-0.304	0.080	-0.407	-0.160
**Individuals and Organizations**				
Politics & Public Authorities	-0.263	0.075	-0.374	-0.144
Fisheries	-0.318	0.084	-0.447	-0.127
Science	-0.305	0.078	-0.414	-0.176
eNGO	-0.384	0.120	-0.517	-0.182
All Groups	-0.314	0.059	-0.389	-0.201

Table S9: Stakeholder shares by groups (multiple occurrences of the same stakeholder per article). The table presents summary statistics for the media coverage of each stakeholder group, accounting for multiple mentions of the same stakeholder within a single article. The calculation is as follows: for each fishery year in the sample, we count the number of mentions of each group (including multiple mentions within one article) and divide by the total number of articles. The table includes the mean, standard deviation (S.D.), minimum (Min.), and maximum (Max.) values for individuals, organizations, and combined individuals and organizations. The stakeholder groups include “Politics & Public Authorities”, “Fisheries”, “Science”, and “eNGO”, as well as a combined "All Groups" category. The values are based on the ‘fishery years’ and are calculated on a yearly basis.

**Individuals**	**Mean**	**S.D.**	**Min.**	**Max.**
Politics & Public Authorities	1.021	0.265	0.558	1.519
Fisheries	1.480	0.306	0.728	2.019
Science	0.531	0.249	0.117	0.938
eNGO	0.271	0.150	0.036	0.498
All Groups	3.303	0.533	2.651	4.283
**Organizations**				
Politics & Public Authorities	1.245	0.263	0.836	1.659
Fisheries	0.567	0.182	0.320	0.807
Science	0.515	0.252	0.214	0.938
eNGO	1.093	0.375	0.618	1.600
All Groups	3.420	0.760	2.136	4.338
**Individuals and Organizations**				
Politics & Public Authorities	2.266	0.381	1.657	2.961
Fisheries	2.047	0.440	1.062	2.802
Science	1.046	0.398	0.330	1.691
eNGO	1.364	0.427	0.823	2.098
All Groups	6.723	1.106	5.010	8.532

Table S10: Stakeholder shares by groups (counting a stakeholder only once per article). The table presents summary statistics for the media coverage of each stakeholder group, counting each stakeholder only once per article, even if mentioned multiple times. The calculation is as follows: for each fishery year in the sample, we count the number of mentions of each group (counting each stakeholder only once per article) and divide by the total number of articles. The table includes the mean, standard deviation (S.D.), minimum (Min.), and maximum (Max.) values for individuals, organizations, and combined individuals and organizations. The stakeholder groups include "Politics & Public Authorities", "Fisheries", "Science", and "eNGO", as well as a combined "All Groups" category. The values are based on the 'fishery years' and are calculated on a yearly basis.

**Individuals**	**Mean**	**S.D.**	**Min.**	**Max.**
Politics & Public Authorities	0.401	0.131	0.209	0.629
Fisheries	0.493	0.130	0.247	0.679
Science	0.177	0,073	0,049	0,313
eNGO	0.134	0.066	0.027	0.244
All Groups	1.205	0.239	0.871	1.571
**Organizations**				
Politics & Public Authorities	0.849	0.172	0.633	1.161
Fisheries	0.436	0.130	0.272	0.685
Science	0.313	0.126	0.155	0.500
eNGO	0.475	0.100	0.327	0.606
All Groups	2.073	0.382	1.524	2.660
**Individuals and Organizations**				
Politics & Public Authorities	1.250	0.262	0.948	1.790
Fisheries	0.929	0.246	0.531	1.364
Science	0.490	0.163	0.233	0.770
eNGO	0.609	0.146	0.355	0.790
All Groups	3.278	0.561	2.563	4.167

Table S11: Newspaper fishery stance by stakeholder group. The table presents summary statistics for the newspaper fishery stance indices for sentences that include a stakeholder belonging to one of the groups. The table includes the mean fishery stance scores, standard deviation (S.D.), minimum (Min.), and maximum (Max.) values for individuals, organizations, and combined individuals and organizations. The stakeholder groups include “Politics & Public Authorities”, “Fisheries”, “Science”, and “eNGO”, as well as a combined "All Groups" category. The fishery stance indices are based on the ‘fishery years’ and are calculated on a yearly basis.

**Individuals**	**Mean**	**S.D.**	**Min.**	**Max.**
Politics & Public Authorities	0.249	0.202	-0.022	0.581
Fisheries	0.460	0.096	0.263	0.559
Science	0.021	0.084	-0.167	0.143
eNGO	-0.119	0.199	-0.600	0.107
All Groups	0.262	0.083	-0.091	0.182
**Organizations**				
Politics & Public Authorities	0.125	0.135	-0.167	0.289
Fisheries	0.594	0.121	0.364	0.766
Science	-0.082	0.085	-0.227	0.085
eNGO	-0.157	0.091	-0.286	0.024
All Groups	0.082	0.083	-0.091	0.184
**Individuals and Organizations**				
Politics & Public Authorities	0.172	0.149	-0.091	0.381
Fisheries	0.499	0.093	0.338	0.608
Science	-0.039	0.075	-0.206	0.089
eNGO	-0.139	0.081	-0.267	-0.020
All Groups	0.170	0.084	-0.0459	0.241

Table S12: Newspaper fishery stance by party. The table presents summary statistics for the newspaper fishery stance indices for sentences that include a political party belonging to one of the groups. The table includes the mean fishery stance scores, standard deviation (S.D.), minimum (Min.), and maximum (Max.) values for individuals, organizations, and combined individuals and organizations. The stakeholder groups include CDU, SPD and Greens, as well as a combined "All Parties" category. The fishery stance indices are based on the ‘fishery years’ and are calculated on a yearly basis.

**Individuals**	**Mean**	**S.D.**	**Min.**	**Max.**
CDU	0.410	0.225	0.000	0.667
SPD	0.413	0.339	-0.364	0.730
Greens	0.069	0.206	-0.250	0.273
All Parties	0.317	0.192	-0.094	0.596
**Organizations**				
CDU	0.367	0.196	0.000	0.621
SPD	0.444	0.314	-0.333	0.778
Greens	-0.029	0.166	-0.300	0.167
All Parties	0.293	0.166	0.000	0.481
**Individuals and Organizations**				
CDU	0.390	0.169	0.100	0.612
SPD	0.422	0.328	-0.350	0.727
Greens	0.038	0.161	-0.267	0.235
All Parties	0.309	0.171	0.000	0.554

3. Supporting Results

3.1. Daily Distribution of Newspaper Articles

The International Council for the Exploration of the Seas (ICES) announces once per year, typically in May, quota recommendations for the EU Commission and Council. The days of these announcements are characterized by a slight and statistically significant increase in articles compared to normal days. Neither of the preceding or following days exhibits any significant difference in publication volume compared to normal days (Figure S5).

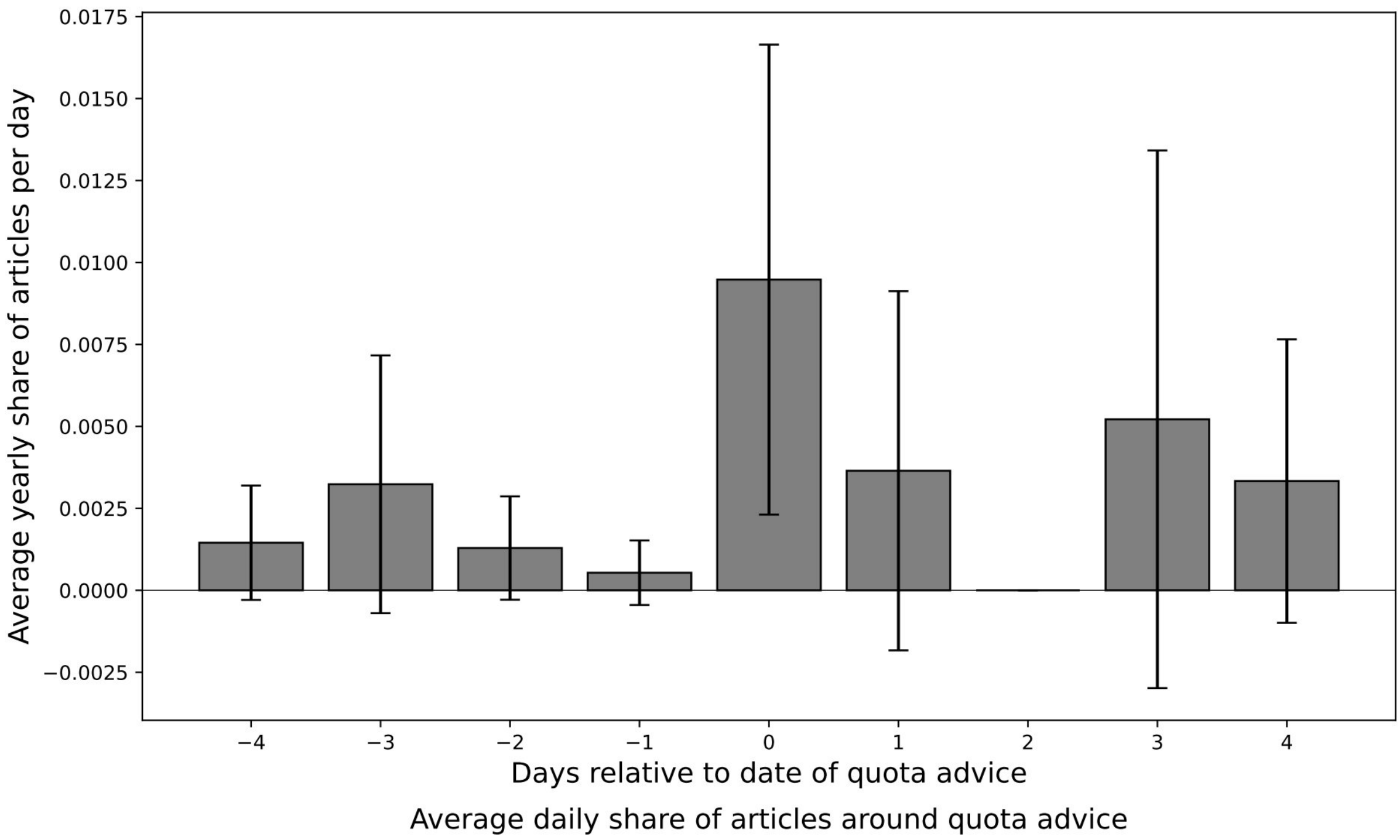

Figure S5: Share of articles around days of quota advice announcements. The bars show the average share of articles around ICES announcement days. We first computed the share of articles published in one of the four days before until four days after an announcement day for each of the years 2009-2021 and then took the sample average of all these daily shares. The gray intervals indicate 95% confidence bounds.

3.2. Baltic Sea fish species in the German media

Fish species diversity in the Western Baltic Sea (WBS) is very low compared to other marine ecosystems such as the North Sea. There are only a few fish species that are traditionally of social and economic importance: herring, cod, salmon, sprat, and plaice (Schwermer et al. 2021; Schacht and Voss 2022). To better understand the salience of each specific fish species for the media discourse, we calculated the shares of articles in our dataset which mention the fish species at least once. To this end, we counted the number of articles each year in which a species was mentioned at least once and divided that by the total number of articles for that year. By conducting this analysis separately for each species, we constructed measures of media attention for herring, cod, salmon, sprat, and plaice.

Our analysis shows that cod and herring have by far the largest media coverage over the sample period 2009 - 2021 (Figure S6). It fluctuates around an upward trend which is particularly pronounced in the second half of the sample. In contrast, media coverage of salmon, sprat and plaice is much smaller and relatively stable over time.

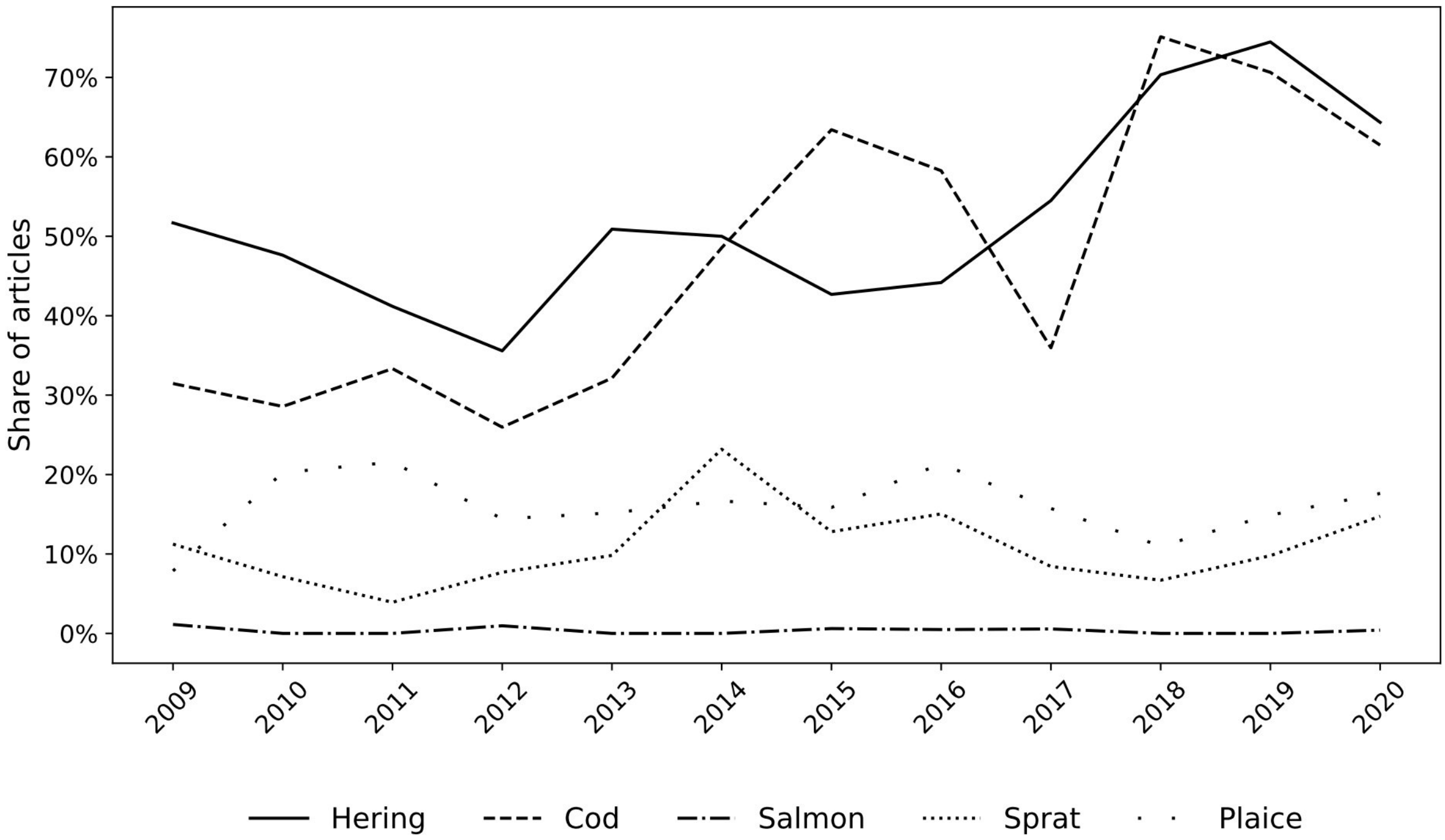

Figure S6: Yearly share of articles for the fish species herring, cod, salmon, sprat and plaice. The shares of given years are calculated by counting share of articles in which the corresponding fish species is mentioned at least once. Years are defined by the calendar year.

The periods 2016 - 2017 as well as 2019 and 2020 are particularly noticeable. While in the first-mentioned period herring demonstrates a 10% higher share of articles compared to cod, in 2019 and 2020 this share is almost equalized (herring in 2019 was at 72.4% and 80.0% in 2020, cod was at 77.4 % in 2019 and 77.3% in 2020). However, the share of articles for herring and cod decreases in 2021 (64.7% for herring and 63.2% for cod). Overall, these results show that herring and cod dominate the media discourse about Germany's Baltic Sea fishery and have done so throughout our entire dataset.

3.3.Word Frequency Analysis

Additionally, we conducted a word frequency analysis to identify the most frequent words occurring in stakeholder-related sentences. To obtain more meaningful results, we excluded all stakeholder names both groups and individuals as well as common stopwords which are words that carry little meaning on their own such as „but“ or „and“.

Our findings that the discourse is divided along commercial fishery on one side and environmental concerns on the other side are further supported by our word frequency analysis. For example, the term “stock” appears frequently in the negative sentences of both Science and eNGOs (Table S15a and Table S15b). Additionally, Science often use the word “crisis” (Table S15a), which indicates a predominant focus on the environmental impact of fisheries. In contrast, words such as “reduction” and “survive” occur more frequently in negative Fishery sentences (Table S15c), highlighting fishers’ concerns about increasing economic instability. Furthermore, in the negative sentences from Politics and Public Authorities, terms like “stock” and “reduction” are common (Table S15d). This suggests that while these groups are mindful of fishers’ interests, there is also a strong emphasis on nature conservation.

Table S15a: Word Count for negative Science sentences. This table shows the 10 most often occurring words in Science sentences with a negative sentiment.

Word	Translation	Count
bestand	stock	56
international	international	54
schlecht	bad	47
empfehlen	recommend	30
empfehlung	recommendation	29
geben	give	27
quote	quota	26
dorsch	cod	26
erklären	explain	25
krise	crisis	25

Table S15b: Word Count for negative eNGO sentences. This table shows the 10 most often occurring words in Science sentences with a negative sentiment.

Word	Translation	Count
kritisieren	criticize	129
bestand	stock	66
nachhaltig	sustainable	49
hoch	high	42

kritik	criticism	38
überfischung	overfishing	38
meer	sea	34
schlecht	bad	33
fordern	demand	32
immer	always	30

Table S15c: Word Count for negative Fishery sentences. This table shows the 10 most often occurring words in Science sentences with a negative sentiment.

Word	Translation	Count
geben	give	54
quote	quote	53
kürzung	reduction	43
betrieb	operation	42
chef	boss	40
sehen	see	38
kritisieren	criticize	34
klein	small	31
überleben	survive	30
problem	problem	30

Table S15d: Word Count for negative Politics and Public Authorities sentences. This table shows the 10 most often occurring words in Science sentences with a negative sentiment.

Word	Translation	Count
bestand	stock	69
sehen	see	54
kürzung	reduction	51
kritisieren	criticize	49
naturschutz	nature conservation	48
bundesamt	federal agency	46
situation	situation	41
vorschlag	proposal	39
drastisch	drastic	39
schwierig	difficult	37

4. Supporting References

Chan, B., S. Schweter, and T. Möller. 2020. German's Next Language Model. In *Proceedings of the 28th International Conference on Computational Linguistics (COLING 2020)*, 6788–6796. https://doi.org/10.18653/v1/2020.coling-main.598

Devlin, J., M.-W. Chang, K. Lee, and K. Toutanova. 2019. BERT: Pre-training of deep bidirectional transformers for language understanding. In *Proceedings of the Conference of the North American Chapter of the Association for Computational Linguistics: Human Language Technologies (NAACL-HLT 2019), Volume 1 (Long and Short Papers),* 4171–4186. https://doi.org/10.18653/v1/N19-1423

EU. 2022. Council Regulation (EU) 2022/2090 of 27 October 2022 fixing the fishing opportunities for certain fish stocks and groups of fish stocks applicable in the Baltic Sea for 2023 and amending Regulation (EU) 2022/109 as regards certain fishing opportunities in other waters. *Official Journal of the European Union*. https://eur-lex.europa.eu/eli/reg/2022/2090/oj/eng

Schacht, K., and R. Voss. 2023. German fishery's adaptation to historic events, Western Baltic Sea, 1890–1950. *Ambio* 52(1): 155–170. https://doi.org/10.1007/s13280-022-01768-2

Schwermer, H., A.M. Blöcker, C. Möllmann, and M. Döring. 2021. The 'Cod-Multiple': Modes of existence of fish, science and people. *Sustainability* 13(21): 12229. https://doi.org/10.3390/su132112229

Vaswani, A., N. Shazeer, N. Parmar, J. Uszkoreit, L. Jones, A.N. Gomez, Ł. Kaiser, and I. Polosukhin. 2017. Attention is all you need. Advances in *Neural Information Processing Systems* 30: 5998–6008. https://doi.org/10.48550/arXiv.1706.03762

Wu, Y., M. Schuster, Z. Chen, Q.V. Le, M. Norouzi, W. Macherey, M. Krikun, and Y. Cao, et al. 2016. Google's neural machine translation system: Bridging the gap between human and machine translation. Preprint at arXiv:1609.08144 [cs.CL]. https://arxiv.org/abs/1609.08144
